# Barriers to cervical cancer screening among refugee women: A systematic review

**DOI:** 10.1371/journal.pgph.0004225

**Published:** 2025-03-18

**Authors:** Md Anwer Hossain, Shimlin Jahan Khanam, Md. Nuruzzaman Khan, John Oldroyd, Rakibul M. Islam

**Affiliations:** 1 Laboratory of Fertility and Well-Being, Max Planck Institute for Demographic Research, Rostock, Germany; 2 School of Geography and Sustainable Development, University of St Andrews, St Andrews, United Kingdom; 3 Department of Population Science, Jatiya Kabi Kazi Nazrul Islam University, Mymensingh, Bangladesh; 4 Nossal Institute for Global Health, Melbourne School of Population and Global Health, The University of Melbourne, Parkville, Victoria, Australia; 5 School of Behavioral and Health Sciences, Australian Catholic University, Fitzroy, Victoria, Australia; 6 South Asian Institute for Social Transformation (SAIST), Dhaka, Bangladesh; 7 School of Public Health and Preventive Medicine, Monash University, Melbourne, Victoria, Australia; University of Greenwich, UNITED KINGDOM OF GREAT BRITAIN AND NORTHERN IRELAND

## Abstract

Cervical cancer disproportionately affects vulnerable populations including refugee women. Understanding the barriers to cervical cancer screening uptake in this group is crucial to inform targeted interventions and improve health outcomes. This review aimed to identify barriers hindering their access to cervical cancer screening. Five databases - Ovid MEDLINE, EMBASE, PsycINFO, CINAHL, and SCOPUS - were searched in December 2024. The inclusion criteria included studies - a) targeting healthy refugee women, b) conducted in community or hospital/clinic settings, c) reporting barriers to cervical cancer screening from the women’s perspective, and d) published in English. Thematic analysis was performed to identify the barriers. The review followed the updated Preferred Reporting Items for Systematic Reviews and Meta-Analyses (PRISMA) guidelines. Eleven studies, seven from the USA, one from each of the UK, Australia, South Korea and Jordan, were included in this review. Of these, six were qualitative, three were quantitative and two were mixed methods studies. There was a consistent pattern of lack of knowledge about cervical cancer and cancer screening in refugee women. A unique barrier was women’s negative experiences in refugee camps. Four interconnected themes emerged including 1) individual level barriers, 2) cultural and religious barriers, 3) social and structural barriers, and 4) healthcare system barriers. Ninety percent of included studies were assessed as medium or high quality. The findings underscore the urgent need for targeted interventions to address the diverse challenges faced by refugee women globally which hinder their access to cervical cancer screening. Strategies should include culturally sensitive awareness campaigns, active engagement of healthcare professionals, and structural reforms within healthcare systems to enhance cervical cancer screening uptake among refugee women.

## Introduction

Cervical cancer is the third most common cancer among women worldwide and the second leading cause of cancer-related mortality among women in low- and middle-income countries (LMICs) [1]. Despite the availability of effective screening methods, cervical cancer remains a leading cause of morbidity and mortality among women globally, with an estimated 604,127 new cases and 341,831 deaths reported in 2020 alone [1]. Cervical cancer can be prevented by regular screening with the Pap smear or the human papillomavirus (HPV) test. The Pap smear detects precancerous changes in the cervix, while the HPV test detects the presence of the HPV virus, which can cause cervical cancer.

Data on cervical cancer-related deaths and disabilities among refugee women remains scarce. This gap is significant because while cervical cancer screening has effectively reduced the incidence and mortality rates among non-refugee women in many developed countries [1], refugee women encounter numerous barriers that hinder their access to and utilization of these critical, life-saving services. Refugee women are a vulnerable group due to their forced displacement, often resulting from conflict, violence, or persecution in their home countries that disrupt their lives, limiting their access to healthcare services and placing them at increased risk of health disparities [2–4]. There is a critical need to comprehensively identify the barriers that impede refugee women’s access to cervical cancer screening services. We hypothesized that refugee women encounter unique barriers that impede cervical cancer screening uptake. This systematic review aimed to synthesize existing literature on these barriers, shedding light on multifaceted obstacles and challenges faced by refugee women, and provide insights to guide the development of targeted interventions aimed at improving cervical cancer screening uptake in this population.

## Materials and methods

This review was conducted according to the updated Preferred Reporting Items for Systematic Reviews and Meta-Analyses (PRISMA) guidelines [5] (S1 PRISMA Checklist).

### Data source and search strategy

A systematic literature search was conducted to identify studies that examined barriers to cervical cancer screening among refugee women using Ovid MEDLINE, EMBASE, PsycINFO, CINAHL, and SCOPUS in December 2023 and updated on 17 December 2024. Both the subject and text searches were done separately combining with ‘OR’ and ‘AND’ operators. The studies were searched using the MeSH (Medical Subject Headings) terms (Table A in S1 Text). Additionally, a retrospective literature search was done to retrieve relevant articles from published papers [6–10].

### Inclusion criteria

Both quantitative and qualitative studies were included to identify the barriers associated with screening uptake. The quantitative studies were analyzed to identify factors related to the uptake of cervical cancer screening. Qualitative studies were included to investigate barriers to cervical cancer screening self-reported by refugee women. The inclusion of qualitative studies in the systematic review also served to triangulate findings from the quantitative studies and provide alternative perspectives [11]. Studies whose target population were healthy refugee women, that were conducted in either community or hospital/clinic settings, and that reported barriers from the women’s perspective only were included in this review. Only English-language studies were included.

### Exclusion criteria

Studies that included respondents with serious illness, that involved the diagnose and/or evaluation of treatment of cervical cancer, and reported barriers from the health care delivery perspective (such as barriers to setting up cytology-based screening programs), were excluded. Studies focusing only on HPV vaccination uptake and migrants, not refugees, were excluded. Studies that reported barrier scores but did not provide data for specific barriers were also excluded. Reviews, editorials, letters, and personal opinions were also excluded.

### Data extraction

Two authors (MAH and SJK) independently extracted the data from the selected papers. Other authors cross-checked all the final papers to ensure accuracy. Conflicts were resolved through discussion with a third reviewer (RMI) before inclusion. We abstracted data into an evidence table and described results in summary format. The following data were extracted for each article: author, year, country, study design and setting, methodology and sampling technique, sample size, respondent’s age group (in years), screening method used, and identified barriers.

### Quality assessment

We used the Critical Appraisal Skills Program (CASP) qualitative checklist [12] and the CASP-modified checklist [12] for their comprehensive, systematic, and widely recognized frameworks for evaluating qualitative and quantitative studies, respectively. The CASP qualitative checklist is well-suited for appraising study rigor and credibility through criteria such as methodology, recruitment, and ethical considerations, aligning with the qualitative nature of some studies in our review. The CASP-modified checklist, tailored for quantitative research, includes additional parameters like statistical validity, representativeness, and local applicability, ensuring thorough and consistent assessment across diverse study designs. These tools’ reliability and adaptability, combined with our scoring and ranking system, facilitated an objective and transparent evaluation of study quality, enhancing rigor and comparability in our systematic review on barriers to cervical cancer screening among refugee women.

The CASP qualitative checklist [12] assesses 10 quality criteria, including clear study objectives, appropriate methodology, appropriate study design, recruitment strategy, data collection, consideration of the relationship between researchers and participants, ethical issues, rigorous analysis, clear findings, and contribution to knowledge. The CASP-modified checklist [12] assesses 9 quality criteria, including clear study objectives, appropriate methodology, representative sample and power, response rate and instrument validation, reliability of the results, appropriate tables and graphs, appropriate statistical methods, important variables considered, and the application of results to local settings. Studies were scored from 0 to 2 for each criterion, with a higher score indicating higher quality. An overall score was calculated as the sum of individual criteria scores. The overall quality of a qualitative study was then ranked as low (0-10), medium (11-15), or high (16-20). The overall quality of a quantitative study was then ranked as low (0-9), medium (10-14), or high (15-18). Two authors (MAH and SJK) independently assessed the quality of the included studies. Any disagreement was resolved by discussion with a third review author (RMI).

### Data synthesis

Since the refugee women came from wide social and cultural contexts, it was imperative to select a framework that incorporated a holistic view of factors that may influence a woman’s cervical cancer screening behavior. Therefore, data was synthesized using *framework synthesis* [13] that includes two processes: 1) developing or selecting an initial framework and 2) recognizing patterns through aggregation.

To recognize the pattern through aggregation, included studies was uploaded to NVivo version 14. Any text that referred to the findings of the included studies were coded and grouped thematically in line with the initial framework. The coded text included participant quotes and interpretive text from the author(s). During this stage, the initial framework was iteratively adapted to reflect the content of the synthesized literature, i.e., by incorporating codes and themes identified during the coding process. Coding and analysis were conducted by a review author (MAH) experienced with qualitative research.

Due to substantial variation in the typology of barriers and the diverse methodologies used in the included studies, results are presented by themes. This analytic approach was undertaken rather than qualitative meta-synthesis due to the small number of studies included in this review.

## Results

### Study characteristics

**Fig 1:**
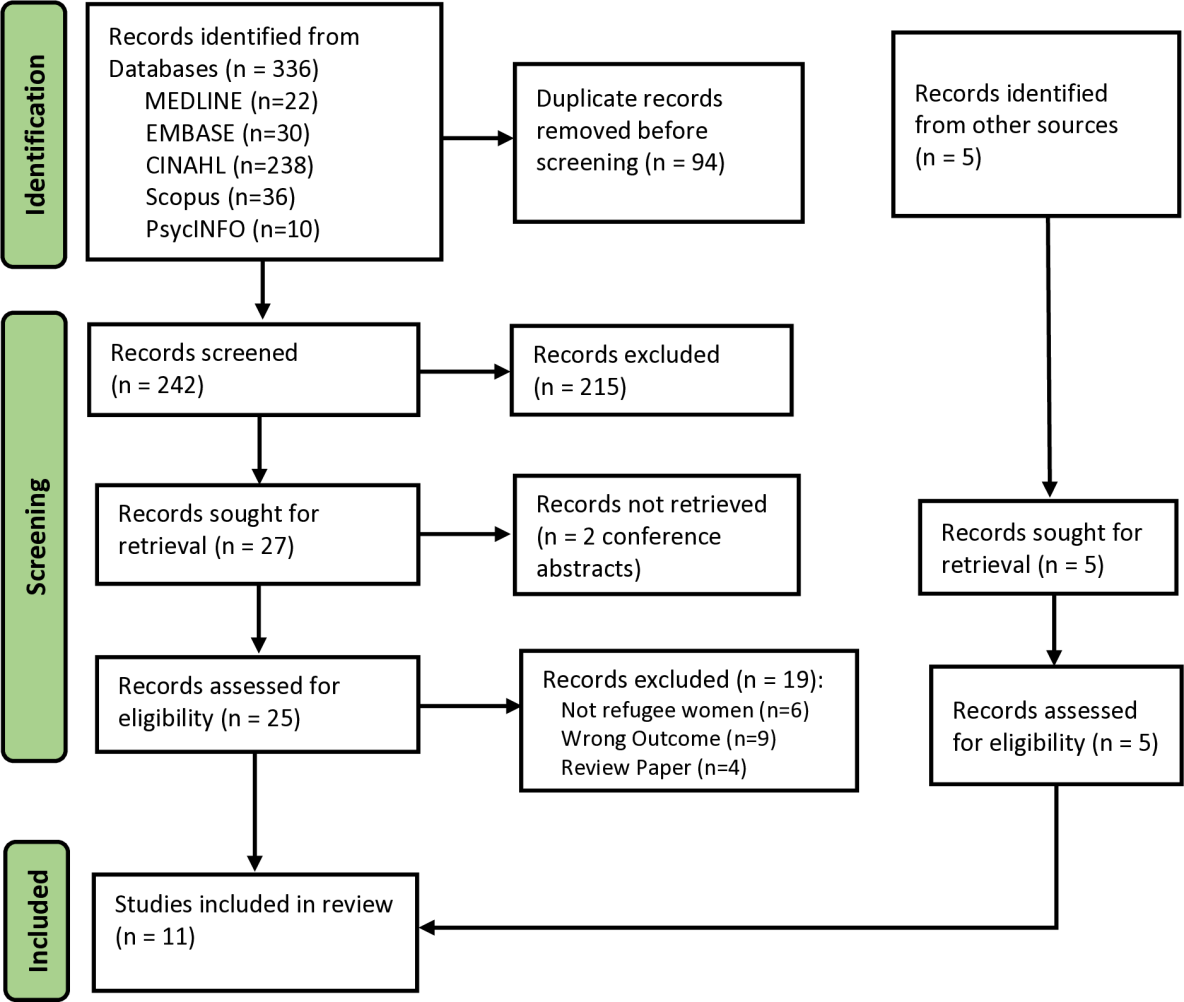
PRISMA flowchart.

The search resulted in 336 studies, of which 94 were duplicates (Fig 1) (Table B in S1 Text). A further 215 papers were excluded after title and abstract screening, leaving 25 studies for full-text review. An additional 5 studies were retrieved from other sources for full-text review. Nineteen full text records were subsequently excluded, and reasons of exclusion are presented in Table C in S1 Text, leaving 11 studies which were included in this review. The included studies were published between 2009 and 2023.

All included studies were cross-sectional in research design. Six were qualitative, three quantitative, and two mixed methods studies. All studies utilized the Pap Smear test or HPV test for cervical cancer screening except one study [14] in which the screening method was not described. Seven studies were conducted in the USA, and one from each of the UK, Australia, South Korea and Jordan. Several barriers to cervical cancer screening among refugee women were identified. These were categorized into themes (Table 1, Table 2).

**Table 1:** Characteristics of included studies.

Author, Year & Country	Study design & Setting	Methodology & Sampling technique	Sample size (n)	Age group (years)	Screening method used	Barriers	Quality rating^*^
Abdullahi et al., 2009 United Kingdom	Cross sectional Community groups, voluntary organizations, mosques and ESOL groups (English classes for speakers of other languages).	Qualitative (Focus group & in-depth interviews) Convenience	50	25-64	Pap smear	Language difficulties Specific cultural issues like fatalistic attitudes, and embarrassment associated with female circumcision, i.e., female genital mutilation Lack of understanding of risk factors for cervical cancer Lack of knowledge about the need for cervical screening The possibility of having a male practitioner perform the test Practical problems such as appointment times and childcare needs, fear of the test and negative past experiences	High
AlAbdulKader et al., 2023 United States	Cross-sectional Federally Qualified Health Center (FQHC) in Cleveland, Ohio, USA	Mixed methods (Electronic medical records & Structured phone-based Survey)	20	21-63	Pap smear/ HPV test	Fear of cancer Language barrier lack of knowledge lack of time Lack of transportation Cost/insurance issues Male doctor Anticipated pain/discomfort	Medium
Allen et al., 2018 United States	Cross-sectional In a multipurpose community center for refugee	Qualitative (Focus group) Convenience	31	23-64	Pap smear	Lack of knowledge about cervical cancer screening and especially about HPV vaccination Hesitation among male partners about screening and HPV vaccination Lack of support from husbands Lack of culturally specific interventions for refugee population	Poor
Elmore et al., 2022, United States	Retrospective electronic medical record review, International Family Medicine Clinic, Central Virginia	Quantitative (Content Analysis) Purposive sampling	525	21-89	Pap smear alone or in combination with HPV testing	Being younger (<30 years) or older (>50 years) Never married Having no children Living in the U.S. for less than 10 years	High
Ghebrendrias et al., 2021 United States	Cross-sectional Among Sub-Saharan Africa and the Middle East refugee through the United Women of East Africa Support Team	Qualitative (Semi-structured & focus group) Convenience	53	20-50	Pap smear	Shyness, fear, confusion, stigma about the test Modesty and cultural barriers Strong preference for female providers Lack of knowledge about cervical cancer screening and Human papilloma virus (HPV) vaccination Language difficulties	Medium
Haworth et al., 2014 United States	Cross-sectional In one city	Mixed method (Survey & focus group) Convenience	Survey = 42 Focus group = 27	19-60	Pap smear	Lack of knowledge about cervical cancer and screening practices Shyness Stigma for going to a male health provider or translator Language difficulties Limited insurance coverage and transportation to health facilities	Medium
Kim et al., 2018 South Korea	Cross-sectional North Korean refugees residing in the Central Seoul area	Qualitative (In-depth interviews) Convenience	8	37-74	Pap smear	Poor knowledge about the screening program Lack of perceived need for preventive services Concerns about costs Anxiety related to having a male healthcare provider	Medium
Kue et al., 2017 United States	Cross sectional In a Midwestern city	Quantitative (Questionnaire survey) Convenience	97	18 or above	Pap smear	Not knowing English Shyness Feeling afraid of having the procedure Pain/discomfort from the procedure Lack of time	Medium
Lor et al., 2018 United States	Cross-sectional Through primary resettlement sites	Qualitative (Focus group) Convenience	31 Burmese and 27 Bhutanese participants	20–65	Pap smear	Misconceptions about cervical cancer screening Barriers to accessing health services include limited English proficiency and problems with interpreters Financial and transportation concerns. Embarrassment and stigma related to cervical cancer and screening	Medium
Muhaidat et al., 2022, Jordan	Cross-sectional Palestinian refugee women residing in the Jerash camp, Jordan	Quantitative (Survey) Convenience	359	19-64	Pap smear	Cost of the examination Being performed by a male doctor Lack of health services nearby Believing that their health is good and they do not need it Fear of knowing the result	Medium
Parajuli et al., 2020 Australia	Cross-sectional In the homes of the study participants	Qualitative (In-depth interviews) Convenience	30	18-23	Cancer screening services (Not specified)	Symptoms-based health-seeking behavior Poor knowledge, awareness Cultural motivations like embarrassment, stigma and refugee camp experiences Health professional behavior Screening not offered, Opportunistic screening without education; and failure to use professional interpreters Changing awareness only among young and educated women but not their caregiver	High

**Table 2:** Thematic classification of the identified barriers to cervical cancer screening in refugee women.

Theme	Barriers
Individual level barriers	• Lack of knowledge about cervical cancer and screening services • Shyness • Anxiety related to having a male healthcare provider • Misconceptions about cervical cancer screening • Lack of perceived need for screening services • Concerns about costs • Lack of time • Fear of the test • Negative past experiences
Cultural and religious barriers	• Stigma for going to a male health provider or translator • Strong preference for female providers • Lack of support from husbands • Lack of culturally specific interventions for the refugee population • Fatalistic attitudes • Modesty and cultural barriers • Embarrassment associated with female circumcision, i.e., female genital mutilation
Social and structural barriers	• Symptoms-based health-seeking behavior • Lack of access to healthcare • Financial and transportation concerns • Poor education • Language barrier • Refugee camp experiences
Healthcare system barriers	• Limited insurance coverage and transportation to health facilities • Health professional behavior (screening not offered, opportunistic screening without education) • Failure to use professional interpreters • Negative attitude among the care-giver • Appointment times and childcare needs

#### Theme 1: Individual level barriers.

Multiple barriers were identified at the individual level, including a lack of knowledge about cervical cancer and screening practices [8,14,15]. Refugee women often lacked awareness of the importance of cervical cancer screening and the procedures involved, leading to a lack of motivation to undergo screening [16–18]. They often were unaware of the need for preventive services, including cervical cancer screening [17]. This may have stemmed from limited access to healthcare services in their home countries or previous experiences where preventive care was not prioritized.

Misconceptions about cervical cancer screening were also prevalent among refugee women [16,18]. These misconceptions included beliefs that screening was unnecessary if no symptoms were present or that the procedure itself was painful or harmful. Fear of the unknown, discomfort during the procedure, and previous negative encounters with healthcare providers influenced their decision to avoid screening [16,19]. Lack of time due to competing responsibilities and priorities also hindered their ability to attend screening appointments [19].

#### Theme 2: Cultural and religious barriers.

Within the cultural and religious context, several barriers were identified. Studies reported that there was a stigma associated with seeking healthcare services from a male provider and/or translator and studies reported that refugee women expressed a strong preference for care from female healthcare providers [7]. Other studies reported that many women expressed discomfort with being examined by a male healthcare provider due to cultural norms and personal preferences [7,15–17]. These preferences were influenced by cultural and religious beliefs that prioritize modesty and gender-specific care. Lack of support from husbands, and the attitudes and opinions of husbands played a significant role in women’s decision-making regarding screening uptake [8,14].

Furthermore, the lack of culturally specific interventions for the refugee population hindered cervical cancer screening uptake [8]. Studies reported that fatalistic attitudes were found among refugee women, leading to a passive approach toward healthcare and preventive services [16]. These attitudes may stem from cultural beliefs or past experiences that have shaped their attitude toward cervical cancer and screening uptake.

#### Theme 3: Social and structural barriers.

Social barriers included symptoms-based health-seeking behavior among refugee women [14]. The included studies reported that refugee women often waited to seek healthcare services when they experienced symptoms only, which results in delayed diagnosis and treatment. Difficulties in reaching healthcare facilities, often exacerbated by geographical remoteness, intensified this challenge [18]. Moreover, transportation issue was an additional barrier that hindered access to cervical cancer screening services, particularly for those residing in remote or underserved areas [15,17,18].

Structural barriers also included poor education among refugee women, which affected their understanding of the importance of cervical cancer screening and their ability to navigate the healthcare system [14,16]. Language barriers further compounded the problem, making it difficult for women to communicate effectively [15–18]. Refugee camp experiences were identified as structural barriers that impacted cervical cancer screening uptake [14]. A unique challenge of refugee camps is the living conditions which often resulted in limited access to healthcare services and a lack of continuity of care.

#### Theme 4: Healthcare system barriers.

Limited insurance coverage to health facilities was a significant health system barrier faced by refugee women [15,18]. This restricted their ability to access screening services, particularly for those are financially not well-off. Failure to offer screening services or provide opportunistic screening without proper education were identified as barriers [14]. Appointment times and childcare needs were practical barriers faced by refugee women [16]. Limited availability of appointments that accommodate their schedules and the need for childcare during screening visits posed challenges to their participation.

### Quality of included studies

The quality of each study is summarized in Table 1. Among the four quantitative studies, only one was rated as high quality, while the remaining three (75%) were medium quality. Among the seven qualitative studies, one (14.3%) study was rated as high and one as poor quality while the rest five (71.4%) were medium quality. The high-quality quantitative study had clear aims, appropriate methods, and local relevance. The high-quality qualitative study also had clear aims, and suitable methods, and had clear descriptions of the value of the research. The details risk-of-bias calculation are presented in Table D and Table E in S1 Text.

## Discussion

To our knowledge, this was the first systematic review of barriers to cervical cancer screening among refugee women. This review demonstrated that a key individual level barrier to screening uptake in refugee women is a lack of knowledge about cervical cancer and/or understanding the importance of cancer screening. Novel findings included women’s anxiety about being examined by male healthcare providers and past refugee camp experiences, both of which served as structural barriers preventing refugee women from seeking cervical cancer screening services. The findings also revealed that refugee women faced multiple interrelated barriers, including socio-cultural, religious, structural, and health system barriers. Cultural and religious barriers included anxiety associated with male healthcare providers, lack of spousal support, and modesty concerns. Social and structural barriers encompassed limited access to healthcare services, financial constraints, low education, language barriers and challenges unique to those with refugee camp experiences. Health system barriers included limited insurance coverage, lack of transportation, unprofessional behavior of healthcare providers, a failure to use professional interpreters, negative caregiver attitudes, and challenges related to appointment times and childcare needs.

The findings of our review were consistent with previous systematic reviews or scoping reviews on barriers to cervical cancer conducted in several ethnic minority and/or underprivileged populations [20–23]. For example, our findings were in line with the findings from a systematic review on barriers to cervical cancer screening uptake in LMICs where lack of knowledge about cervical cancer and understanding of the role of screening were key barriers [24]. We also found that language and communication barriers were a major obstacle to cervical cancer screening among refugee women. Refugee women often had low levels of literacy in both English and their native languages, which affected their access to health information and services, especially for those who had recently resettled [15]. Our systematic review found that emotional barriers, perceived stigma and family dynamics influenced screening participation which is consistent with the findings of a qualitative systematic review on underserved women [25]. Our review was congruent with a scoping review on immigrant Muslim women globally [23]. However, this study also revealed some novel findings, including women’s anxiety about using male healthcare providers, and specific social and structural barriers for those with refugee camp experiences. These findings highlight the universality of challenges faced by refugee women across diverse settings.

Our systematic review also highlighted how the experiences of refugee women may differ from other groups of women. For instance, we found that refugee women experienced difficulties accessing healthcare facilities due to geographical remoteness and lack of transportation. Many refugee women also reported negative past experiences with healthcare providers, such as sexual assault, female genital mutilation, or unprofessional behavior that reduced their trust in healthcare services. These adverse experiences of refugee women underscore the need for greater access to health care services as well as care that considers their diverse needs and backgrounds.

## Strengths and limitations

The major strength of this study is that we conducted a comprehensive literature search across five databases and used a systematic and rigorous approach to appraise the quality of the included studies to synthesize the results. This enhanced the validity and reliability of the findings. However, we were unable to control for the contextual differences among the refugee settings in different countries concerning their legal, political, economic, and health system factors, which may influence the patterns of health care service utilization. Therefore, the findings of this study may not be generalizable to all refugee settings or populations. The review also lacked quantitative data in the included studies which precluded conducting meta-analysis. The findings of this review should be interpreted with a caution since all quantitative studies neither reported response rates and/nor used validated questionnaires, while in the assessment of the quality of all qualitative studies, poor value of the research was present because they contributed poorly to the existing knowledge.

## Conclusion and policy implications

This systematic review demonstrated that refugee women faced multiple interrelated barriers to cervical cancer screening, including their unique experiences in refugee camps. The findings suggest that interventions should aim to increase the awareness and knowledge of cervical cancer and its screening services among refugee women. Furthermore, interventions should be tailored to the diverse needs and preferences of refugee women which consider their cultural and religious backgrounds, language, and literacy levels. Such interventions should promote a trusting and respectful relationship between refugee women and healthcare providers while ensuring the confidentiality and privacy of their information, thereby enhancing cervical cancer screening uptake among refugee women.

## Supporting information

S1 Text Tables. (DOCX)

S1 Checklist PRISMA checklist. (DOCX)
