# Correction: Barriers to cervical cancer screening among refugee women: A systematic review

**DOI:** 10.1371/journal.pgph.0005060

**Published:** 2025-08-12

**Authors:** Md Anwer Hossain, Shimlin Jahan Khanam, Md. Nuruzzaman Khan, John Oldroyd, Rakibul M. Islam

In the Results section, there is a missing subheading before Theme 1: Individual level barriers. The correct subheading is: Barriers to cervical cancer screening among refugee women.

S1 Text was uploaded incorrectly. Please view the correct S1 Text file below.

## Supporting information

S1 Text Tables. (DOCX)
