# Supplementary material for: Barriers to cervical cancer screening among refugee women: A systematic review
Source: PLOS Glob Public Health. 2025 Mar 18;5(3):e0004225. doi: 10.1371/journal.pgph.0004225 (PMC11918313; doi:10.1371/journal.pgph.0004225)
Supplement: S1 Text — (DOCX) [file pgph.0004225.s001.docx]

**Table A: Search Strategy**

**Database 1: Ovid MEDLINE 1946 to 17 December 2024**

| **#** | **Searches** | **Results** |
| --- | --- | --- |
| 1 | exp Refugees/ or exp asylum seekers/ | 14333 |
| 2 | (refugee* or asylum seeker*).mp. | 20876 |
| 3 | 1 or 2 | 20876 |
| 4 | (female* or wom#n or girl*).mp. | 10542757 |
| 5 | exp Cervix Uteri/ | 29900 |
| 6 | cervical.mp. | 318192 |
| 7 | Neoplasms/ | 533574 |
| 8 | (cervix* adj3 neoplas*).mp. | 6512 |
| 9 | 5 or 6 or 7 or 8 | 850453 |
| 10 | exp Mass Screening/ | 149014 |
| 11 | early detection of cancer.mp. | 44539 |
| 12 | papanicolaou.mp. or exp "Early Detection of Cancer"/ | 52372 |
| 13 | human papillomavirus.mp. or exp Alphapapillomavirus/ | 51181 |
| 14 | 10 or 11 or 12 or 13 | 235818 |
| 15 | barrie*.mp. | 478222 |
| 16 | obstacl*.mp. | 74286 |
| 17 | challeng*.mp. | 1389734 |
| 18 | 15 or 16 or 17 | 1864580 |
| 19 | 3 and 4 and 9 and 14 and 18 | 22 |

**Database 2: Embase 1947 to 17 December 2024**

| **#** | **Searches** | **Results** |
| --- | --- | --- |
| 1 | exp Refugees/ or exp asylum seekers/ | 19736 |
| 2 | (refugee* or asylum seeker*).mp. | 24319 |
| 3 | 1 or 2 | 24319 |
| 4 | (female* or wom#n or girl*).mp. | 13651790 |
| 5 | exp Cervix Uteri/ | 38519 |
| 6 | cervical.mp. | 440495 |
| 7 | Neoplasms/ | 82327 |
| 8 | (cervix* adj3 neoplas*).mp. | 4130 |
| 9 | 5 or 6 or 7 or 8 | 541070 |
| 10 | exp Mass Screening/ | 346402 |
| 11 | early detection of cancer.mp. | 4184 |
| 12 | papanicolaou.mp. or exp "Early Detection of Cancer"/ | 42920 |
| 13 | human papillomavirus.mp. or exp Alphapapillomavirus/ | 65518 |
| 14 | 10 or 11 or 12 or 13 | 433807 |
| 15 | barrie*.mp. | 630375 |
| 16 | obstacl*.mp. | 91378 |
| 17 | challeng*.mp. | 1722329 |
| 18 | 15 or 16 or 17 | 2345542 |
| 19 | 3 and 4 and 9 and 14 and 18 | 30 |

**Database 3: APA PsycInfo 1806 to 17 December 2024**

| **#** | **Searches** | **Results** |
| --- | --- | --- |
| 1 | exp Refugees/ or exp asylum seekers/ | 9802 |
| 2 | (refugee* or asylum seeker*).mp. | 14484 |
| 3 | 1 or 2 | 14484 |
| 4 | (female* or wom#n or girl*).mp. | 1391046 |
| 5 | exp Cervix Uteri/ | 0 |
| 6 | cervical.mp. | 10013 |
| 7 | Neoplasms/ | 46947 |
| 8 | (cervix* adj3 neoplas*).mp. | 2 |
| 9 | 5 or 6 or 7 or 8 | 54916 |
| 10 | exp Mass Screening/ | 0 |
| 11 | early detection of cancer.mp. | 1867 |
| 12 | papanicolaou.mp. or exp "Early Detection of Cancer"/ | 640 |
| 13 | human papillomavirus.mp. or exp Alphapapillomavirus/ | 2421 |
| 14 | 10 or 11 or 12 or 13 | 4572 |
| 15 | barrie*.mp. | 110606 |
| 16 | obstacl*.mp. | 24947 |
| 17 | challeng*.mp. | 337602 |
| 18 | 15 or 16 or 17 | 446716 |
| 19 | 3 and 4 and 9 and 14 and 18 | 10 |

**Database 4: CINHAL**

| **ID #** | **Search Terms** | **Search Options** | **Actions** |
| --- | --- | --- | --- |
| S5 | S1 AND S2 AND S3 AND S4 | Expanders - Apply equivalent subjects  Search modes - Boolean/Phrase | View Results (238)  View Details |
| S4 | TX barrie* OR TX obstacl* OR TX challeng* | Expanders - Apply equivalent subjects  Search modes - Boolean/Phrase | View Results (943,125)  View Details |
| S3 | MH (Mass Screening OR Early Detection of Cancer ) OR TX early detection of cancer OR TX human papillomavirus OR TX Alphapapillomavirus | Expanders - Apply equivalent subjects  Search modes - Boolean/Phrase | View Results (43,900)  View Details |
| S2 | MH ( Cervix Uteri* OR Neoplasms ) OR TX cervical OR TX cervix* adj3 neoplas* | Expanders - Apply equivalent subjects  Search modes - Boolean/Phrase | View Results (205,618)  View Details |
| S1 | (MH Refugees OR MH (asylum seekers) OR TX refugee* OR TX (asylum seekers) ) | Expanders - Apply equivalent subjects  Search modes - Boolean/Phrase | View Results (23,589)  View Details |

**Database 5: Scopus**

| **Search** | **Citation** |
| --- | --- |
| ( TITLE-ABS-KEY ( *refugees*  OR  *refugee** OR asylum seeker*)  AND  TITLE-ABS-KEY ( ( *female**  OR  *wom#n*  OR  *girl** ) )  AND  TITLE-ABS-KEY ( ( *cervix*  AND  *uteri* )  OR  *cervical*  OR  *neoplasms*  OR  ( *cervix**  AND  *adj3*  AND  *neoplas** ) )  AND  TITLE-ABS-KEY ( *screening*  OR  ( *mass*  AND  *screening* )  OR  ( *early*  AND  *detection*  AND  *of*  AND  *cancer* )  OR  ( *pap*  AND  *smear* )  OR  *papanicolaou*  OR  *hpv*  OR  ( *human*  AND  *papillomavirus* ) )  AND  TITLE-ABS-KEY ( *barrie**  OR  *challeng**  OR  *obstacl** ) ) | **36** |

**Table B: List of Screened Studies (n=242)**

| **Sl** | **Screened Literature** | **Status^*^** | **Reason for excluding** |
| --- | --- | --- | --- |
| 1 | Abboud, S., E. De Penning, B. M. Brawner, U. Menon, K. Glanz and M. S. Sommers (2017). "Cervical Cancer Screening Among Arab Women in the United States: An Integrative Review." Oncology Nursing Forum 44(1): E20-E33. | R | Not Refugee Women |
| 2 | Abdi, H. I., E. Hoover, S. E. Fagan and P. Adsul (2020). "Cervical Cancer Screening Among Immigrant and Refugee Women: Scoping-Review and Directions for Future Research." Journal of immigrant and minority health 22(6): 1304-1319. | R | Review Paper |
| 3 | Adegboyega, A. O. and J. Hatcher (2016). "Unequal Access: African Immigrants and American Health Care." Kentucky Nurse 64(1): 10-12. | R | Not Refugee Women |
| 3 | Ahc, M. (2021). "Researchers Study Immigrant Latinas' Experience with Reproductive Healthcare: Language barrier is prominent obstacle." Contraceptive Technology Update 43(3): 1-3. | R | Not Refugee Women |
| 4 | Al Qadire, M. I. and K. Alomari (2020). "Syrian Refugees in Jordan: Barriers to receiving optimal cancer care." Clinical Journal of Oncology Nursing 24(6): 707-710. | R | Wrong Outcome |
| 5 | Alam, Z., J. M. Cairns, M. Scott, J. A. Dean and M. Janda (2023). "Interventions to increase cervical screening uptake among immigrant women: A systematic review and meta-analysis." PloS one 18(6): e0281976. | R | Review Paper |
| 6 | Al-Amoudi, S., J. Cañas, S. D. Hohl, S. R. Distelhorst and B. Thompson (2015). "Breaking the Silence: Breast Cancer Knowledge and Beliefs Among Somali Muslim Women in Seattle, Washington." Health Care for Women International 36(5): 608-616. | R | Wrong Outcome |
| 7 | Allaire, B. T., D. Ekweme, T. J. Hoerger, A. DeGroff, S. H. Rim, S. Subramanian and J. W. Miller (2019). "Cost-effectiveness of patient navigation for breast cancer screening in the National Breast and Cervical Cancer Early Detection Program." Cancer Causes & Control 30(9): 923-929. | R | Wrong Outcome |
| 8 | Allen, E. M., H. Y. Lee, R. Pratt, H. Vang, J. R. Desai, A. Dube and E. Lightfoot (2018). "Facilitators and Barriers of Cervical Cancer Screening and Human Papilloma Virus Vaccination Among Somali Refugee Women in the United States: A Qualitative Analysis." Journal of transcultural nursing: official journal of the Transcultural Nursing Society 30(1): 55-63. | A | - |
| 9 | Allen, E. M. and J. O. Loftus (2016). "Barriers to recommended screening among U.S. immigrants: A literature review." Cancer Epidemiology Biomarkers and Prevention 25(3 Supplement). | R | Review Paper |
| 10 | Alomar, A. Z. (2021). "Confidence level, challenges, and obstacles faced by orthopedic residents in obtaining informed consent." Journal of Orthopaedic Surgery & Research 16(1): 1-13. | R | Wrong Outcome |
| 11 | Anaman-Torgbor, J. A., J. King and I. Correa-Velez (2017). "Barriers and facilitators of cervical cancer screening practices among African immigrant women living in Brisbane, Australia." European journal of oncology nursing : the official journal of European Oncology Nursing Society 31: 22-29. | R | Not Refugee Women |
| 12 | Appel, H. and K. Cook (2017). "Cancer screening for immigrants and refugees- a cultural and linguistic approach." Journal of Women's Health 26(4): A4. | R | Wrong Outcome |
| 13 | Arnsberger, P., B. Nussey, P. Fox and W. Breuer (2002). "Cervical intraepithelial lesions and cervical cancer among Asian Pacific Islander women in a cervical cancer screening program." Health Care for Women International 23(5): 450-459. | R | Not Refugee Women |
| 14 | Avieli, H., T. B. Winterstein and T. Gal (2021). "Challenges in Implementing Restorative Justice with Older Adults: Institutional Gatekeepers and Social Barriers." British Journal of Social Work 51(4): 1445-1462. | R | Not Refugee Women |
| 15 | Azzari, C., J. Diez-Domingo, E. Eisenstein, S. N. Faust, A. Konstantopoulos, G. S. Marshall, F. Rodrigues, T. F. Schwarz and C. Weil-Olivier (2020). "Experts' opinion for improving global adolescent vaccination rates: a call to action." European Journal of Pediatrics 179(4): 547-553. | R | Not Refugee Women |
| 16 | Baker, D. L., M. T. Dang, M. Y. Ly and R. Diaz (2010). "Perception of barriers to immunization among parents of Hmong origin in California." American Journal of Public Health 100(5): 839-845. | R | Not Refugee Women |
| 17 | Bang, H. N., S. L. Stewart, T. N. Tung, B.-T. Ngoc and S. J. McPhee (2015). "Effectiveness of Lay Health Worker Outreach in Reducing Disparities in Colorectal Cancer Screening in Vietnamese Americans." American Journal of Public Health 105(10): 2083-2089. | R | Wrong Outcome |
| 18 | Bannour, R., Z. Harzali, W. Dhouib, A. Ben Cheikh, S. Bhiri, H. Ghali, S. Khefacha, H. Said Latiri and M. Ben Rejeb (2021). "Social support and health-related quality of life among patients with chronic heart failure...14th European Public Health Conference (Virtual), Public health futures in a changing world, November 10-12, 2021." European Journal of Public Health 31: iii567-iii567. | R | Not Refugee Women |
| 19 | Barnack-Tavlaris, J. L., L. M. Garcini, C. A. Macera, S. Brodine and E. A. Klonoff (2016). "Human Papillomavirus Vaccination Awareness and Acceptability Among U.S.-Born and U.S. Foreign-Born Women Living in California." Health Care for Women International 37(4): 444-462. | R | Not Refugee Women |
| 20 | Bartlett, R., T. Robinson, J. Anand, F. Negussie, J. Simons Smith and J. A. Boyle (2022). "Empathy and journey mapping the healthcare experience: a community-based participatory approach to exploring women’s access to primary health services within Melbourne’s Arabic-speaking refugee communities." Ethnicity and Health 27(3): 584-600. | R | Wrong Outcome |
| 21 | Beckett, M. (2016). "The Borders that Remain: Prevention of Cervical Cancer in Refugee and Immigrant Women in Canada." UOJM 6(2). | R | Not Refugee Women |
| 22 | Belinski, D. E. and Z. Kanji (2018). "Intersections between clinical dental hygiene education and perceived practice barriers." Canadian Journal of Dental Hygiene 52(2): 132-139. | R | Wrong Outcome |
| 23 | Bernath, B. and Z. Kanji (2021). "Exploring barriers to oral health care experienced by individuals living with autism spectrum disorder." Canadian Journal of Dental Hygiene 55(3): 160-166. | R | Wrong Outcome |
| 24 | Bhadani, U. (2021). "Oral Presentation." Indian Journal of Palliative Care 27(3): 453-470. | R | Wrong Outcome |
| 25 | Bhatta, M. P., D. C. Johnson, M. Lama, B. Maharjan, P. Lhaki and S. Shrestha (2020). "Cervical Cancer and Human Papillomavirus Vaccine Awareness Among Married Bhutanese Refugee and Nepali Women in Eastern Nepal...International Papillomavirus Society Conference in Cape Town, South Africa, February 28–March 4, 2017." Journal of Community Health 45(3): 516-525. | R | Wrong Outcome |
| 26 | Boehnlein, T., J. Kretschmar, W. Regoeczi and J. Smialek (2020). "Responding to Stalking Victims: Perceptions, Barriers, and Directions for Future Research." Journal of Family Violence 35(7): 755-768. | R | Wrong Outcome |
| 27 | Burke, N. J., H. H. Do, J. Talbot, C. Sos, S. Ros and V. M. Taylor (2015). "Protecting our Khmer daughters: ghosts of the past, uncertain futures, and the human papillomavirus vaccine." Ethnicity & Health 20(4): 376-390. | R | Wrong Outcome |
| 28 | Butcher, B. W., T. L. Eaton, A. A. Montgomery-Yates and C. M. Sevin (2022). "Meeting the Challenges of Establishing Intensive Care Unit Follow-up Clinics." American Journal of Critical Care 31(4): 324-328. | R | Wrong Outcome |
| 29 | Castillo, Y. A., K. Rinehart, J. Fischer and W. Weber (2021). "Strategies and Barriers to Work Behavior Changes: Perceptions of Prevocational Rehabilitation Professionals." Journal of Applied Rehabilitation Counseling 52(3): 213-231. | R | Wrong Outcome |
| 30 | Caulfield, L. (2021). "A literature review exploring the perceived impact, challenges and barriers of advanced and consultant practice in therapeutic radiography." Radiography 27(3): 950-955. | R | Review Paper |
| 31 | Černý, V., M. Maegele, V. Agostini, D. Fries, S. R. Leal-Noval, G. Nardai, G. Nardi, A. Östlund and H. Schöchl (2022). "Variations and obstacles in the use of coagulation factor concentrates for major trauma bleeding across Europe: outcomes from a European expert meeting." European Journal of Trauma & Emergency Surgery 48(2): 763-774. | R | Wrong Outcome |
| 32 | Chen, W.-T., B. Guthrie, C.-S. Shiu, J. P. Yang, Z. Weng, L. Wang, E. Kamitani, Y. Fukuda and B. V. Luu (2014). "Acculturation and perceived stress in HIV+ immigrants: depression symptomatology in Asian and Pacific Islanders." AIDS Care 26(12): 1581-1585. | R | Wrong Outcome |
| 33 | Chongsuwat, T., E. R. Decker, M. Wilde, M. B. Fitzpatrick and M. Moua (2024). "Development of Cervical Cancer Prevention Workshops for Hmong and Karenni Women Through a Community-Academic Partnership." WMJ : official publication of the State Medical Society of Wisconsin 123(5): 339-343. | R | Not Refugee Women |
| 34 | Chowdhury, N., J. Naidu, M. Z. I. Chowdhury, M. Vaska, N. Rumana, M. A. A. Lasker and T. C. Turin (2021). "Knowledge translation in health and wellness research focusing on immigrants in Canada." Journal of Primary Health Care 13(2): 139-156. | R | Wrong Outcome |
| 35 | Church, S., S. Ejder Apay, A. Gurol, Y. Slaveva and R. Mills (2023). "Student midwives' perspectives of women's sexual and reproductive health literacy in Turkey." Sexual and Reproductive Healthcare 37: 100864. | R | Wrong Outcome |
| 36 | Cofie, L. E., J. M. Hirth and R. Wong (2018). "Chronic comorbidities and cervical cancer screening and adherence among US-born and foreign-born women." Cancer Causes & Control 29(11): 1105-1113. | R | Not Refugee Women |
| 37 | Connolly, C., D. Wilson, R. Missett, W. C. Dooley, P. A. Avent and R. Wright (2004). "Academic practice exemplars. Associate degree nursing in a community-based health center network: lessons in collaboration." Journal of Nursing Education 43(2): 78-80. | R | Wrong Outcome |
| 38 | Crawford, J., F. Ahmad, D. Beaton and A. S. Bierman (2016). "Cancer screening behaviours among South Asian immigrants in the UK, US and Canada: a scoping study." Health & Social Care in the Community 24(2): 123-153. | R | Review Paper |
| 39 | Cudjoe, J., R.-A. Turkson-Ocran, A. K. Ezeigwe, Y. Commodore-Mensah, M. Nkimbeng and H.-R. Han (2019). "Recruiting African Immigrant Women for Community-Based Cancer Prevention Studies: Lessons Learned from the AfroPap Study." Journal of Community Health 44(5): 1019-1026. | R | Not Refugee Women |
| 40 | Daponte, A., M. Bernal, J. Bolívar, I. Mateo, L.-R. Salmi, S. Barsanti, L. Berghmans, E. Piznal, Y. Bourgueil, S. Marquez, I. González, A. Carriazo, Z. Maros-Szabo and S. Ménival (2014). "Criteria for implementing interventions to reduce health inequalities in primary care settings in European regions." European Journal of Public Health 24(6): 979-989. | R | Wrong Outcome |
| 41 | Davidson, N., K. Hammarberg, L. Romero and J. Fisher (2022). "Access to preventive sexual and reproductive health care for women from refugee-like backgrounds: a systematic review." BMC Public Health 22(1). | R | Review Paper |
| 42 | Davies, G., N. Mills, C. Holcombe, S. Potter, B. R. A. S. G. on behalf of the i, N. L. P. Barnes, J. M. Blazeby, O. A. Branford, R. I. Cutress, M. D. Gardiner, C. Holcombe, A. Jain, K. McEvoy, N. Mills, S. Mylvaganam, S. Potter, J. M. Skillman, E. M. Teasdale, S. Thrush and Z. Tolkien (2020). "Perceived barriers to randomised controlled trials in breast reconstruction: obstacle to trial initiation or opportunity to resolve? A qualitative study." Trials 21(1): 1-11. | R | Wrong Outcome |
| 43 | de Queiroz, D. M., L. Conde de Oliveira, P. A. de Araújo Filho and M. R. Ferreira da Silva (2021). "Challenges and potentials of the production of comprehensive care in Primary Health Care in Brazil." Revista Brasileira de Enfermagem 74(5): 1-10. | R | Wrong Outcome |
| 44 | Dönmez, S., R. Öztürk, S. Kısa, B. Karaoz Weller and S. Zeyneloğlu (2019). "Knowledge and perception of female nursing students about human papillomavirus (HPV), cervical cancer, and attitudes toward HPV vaccination." Journal of American College Health 67(5): 410-417. | R | Not Refugee Women |
| 45 | Douglass, K., L. Narayan, R. Allen, J. Pandya and Z. Talib (2021). "Language diversity and challenges to communication in Indian emergency departments." International Journal of Emergency Medicine 14(1): 1-8. | R | Wrong Outcome |
| 46 | Egli-Gany, D., W. Aftab, S. Hawkes, L. Abu-Raddad, K. Buse, F. Rabbani, N. Low and K. Onarheim (2021). "The social and structural determinants of sexual and reproductive health and rights in migrants and refugees: a systematic review of reviews." Eastern Mediterranean Health Journal 27(12): 1203-1213. | R | Review Paper |
| 47 | Elewonibi, B. and R. BeLue (2019). "The influence of socio-cultural factors on breast cancer screening behaviors in Lagos, Nigeria." Ethnicity & Health 24(5): 544-559. | R | Wrong Outcome |
| 48 | Ethier, A. and A. Carrier (2021). "A Scoping Review of the Implementation of Local Health and Social Services for Older Adults." Healthcare Policy 17(2): 105-118. | R | Review Paper |
| 49 | Fadhil, I., E. Alkhalawi, R. Nasr, H. Fouad, P. Basu, R. Camacho and H. Alsaadoon (2021). "National cancer control plans across the Eastern Mediterranean region: challenges and opportunities to scale-up." Lancet Oncology 22 1077-4114 (Print)(11): e517-e529. | R | Wrong Outcome |
| 50 | Faguy, K. (2020). "Challenges of the Underserved and Underscreened in Mammography." Radiologic Technology 91(3): 267M-283M. | R | Wrong Outcome |
| 51 | Fang, D. M. and D. L. Baker (2013). "Barriers and facilitators of cervical cancer screening among women of Hmong origin." Journal of health care for the poor and underserved 24(2): 540-555. | R | Not Refugee Women |
| 52 | Fang, D. M. and D. L. Baker (2013). "Barriers and facilitators of cervical cancer screening among women of Hmong origin." J Health Care Poor Underserved 24(2): 540-555. | R | Not Refugee Women |
| 53 | Ferdous, M., S. Lee, S. Goopy, H. Yang, N. Rumana, T. Abedin and T. C. Turin (2018). "Barriers to cervical cancer screening faced by immigrant women in Canada: a systematic scoping review." BMC Women's Health 18(1): N.PAG-N.PAG. | R | Review Paper |
| 54 | Forwood, L. (2014). "Preventing cervical cancer in Karen women-A peer education project." Asia-Pacific Journal of Clinical Oncology 10(SUPPL. 9): 18-19. | R | Wrong Outcome |
| 55 | Gagliardi, A. R., C. Kim and B. Jameel (2020). "Physician behaviours that optimize patient‐centred care: Focus groups with migrant women." Health Expectations 23(5): 1280-1288. | R | Wrong Outcome |
| 56 | Galanis, P., S. Koureas, O. Siskou, O. Konstantakopoulou, G. Angelopoulos and D. Kaitelidou (2022). "Healthcare Services Access, Use and Barriers among Migrants in Europe: A Systematic Review." International Journal of Caring Sciences 15(1): 28-47. | R | Wrong Outcome |
| 57 | Gany, F. M., A. P. Herrera, M. Avallone and J. Changrani (2006). "Attitudes, knowledge, and health-seeking behaviors of five immigrant minority communities in the prevention and screening of cancer: a focus group approach." Ethnicity & Health 11(1): 19-39. | R | Not Refugee Women |
| 58 | Gea‐Sánchez, M., D. Gastaldo, F. Molina‐Luque and L. Otero‐García (2017). "Access and utilisation of social and health services as a social determinant of health: the case of undocumented Latin American immigrant women working in Lleida ( Catalonia, Spain)." Health & Social Care in the Community 25(2): 424-434. | R | Not Refugee Women |
| 59 | Ghebrendrias, S., S. Pfeil, B. Crouthamel, M. Chalmiers, G. Kully and S. Mody (2021). "An Examination of Misconceptions and Their Impact on Cervical Cancer Prevention Practices among Sub-Saharan African and Middle Eastern Refugees." Health Equity 5(1): 382-389. | A | - |
| 60 | Ghirimoldi, F. and G. Sanchez-Soto (2021). "Immigrant assimilation and profiles of breast cancer screening behaviors among U.S. immigrant women." Health Care for Women International 42(2): 213-234. | R | Not Refugee Women |
| 61 | Gomez, S. L., J. L. Kelsey, S. L. Glaser, M. M. Lee and S. Sidney (2004). "Immigration and acculturation in relation to health and health-related risk factors among specific Asian subgroups in a health maintenance organization." American Journal of Public Health 94(11): 1977-1984. | R | Not Refugee Women |
| 62 | Gonçalves do Nascimento, S., C. P. Andra de Lima de Carvalho, R. Soares da Silva, C. M. de Oliveira and C. Vieira do Bonfim (2018). "Decline of mortality from cervical cancer." Revista Brasileira de Enfermagem 71: 585-590. | R | Wrong Outcome |
| 63 | Gonzalez, T., R. Harris, R. Williams, R. Wadwell, K. Barlow‐Stewart, J. Fleming and M. Buckman (2020). "Exploring the barriers preventing Indigenous Australians from accessing cancer genetic counseling." Journal of Genetic Counseling 29(4): 542-552. | R | Not Refugee Women |
| 64 | Gorin, S. (2005). "Correlates of Colorectal Cancer Screening Compliance Among Urban Hispanics." Journal of Behavioral Medicine 28(2): 125-137. | R | Wrong Outcome |
| 65 | Guilfoyle, S., R. Franco and S. S. Gorin (2007). "Exploring older women's approaches to cervical cancer screening." Health Care for Women International 28(10): 930-950. | R | Not Refugee Women |
| 66 | Hanna, R. M., M. Troyer and T. D. Bui (2015). "Delivering healthcare to the refugee population in Pittsburgh." Annals of Global Health 81(1): 221-222. | R | Wrong Outcome |
| 67 | Haworth, R. J., R. Margalit, C. Ross, T. Nepal and A. S. Soliman (2014). "Knowledge, attitudes, and practices for cervical cancer screening among the Bhutanese refugee community in Omaha, Nebraska." Journal of community health 39(5): 872-878. | A |  |
| 68 | Hickey, J. E., A. J. Gagnon and N. Jitthai (2016). "Knowledge about pandemic influenza preparedness among vulnerable migrants in Thailand." Health Promotion International 31(1): 124-132. | R | Wrong Outcome |
| 69 | Hohman, K., L. Given, L. Graaf, J. Sergeant, D. Muthukuda, T. Devery, K. Jones and K. W. Sittig (2018). "Evolution of comprehensive cancer control plans and partnerships." Cancer Causes & Control 29(12): 1181-1193. | R | Not Refugee Women |
| 70 | Huhmann, K. (2020). "Barriers and Facilitators to Breast and Cervical Cancer Screening in Somali Immigrant Women: An Integrative Review." Oncology Nursing Forum 47(2): 177-186. | R | Review Paper |
| 71 | Hussain, Z., A. Y. Barden-Maja and J. Michener (2016). "Cervical cancer education and screening in women's refugee clinic: The role of in-person interpreters." Journal of General Internal Medicine 31(2 SUPPL. 1): S867. | R | Wrong Outcome |
| 72 | Hynes, L. (2021). "Intensive care nurses' perceptions on barriers impeding the provision of end of life care in the intensive care setting: a quantitative analysis." Australian Journal of Advanced Nursing 39(1): 7-17. | R | Wrong Outcome |
| 73 | Ibrahim, E., N. Hamed and L. Ahmed (2021). "Views of primary health care providers of the challenges to screening for intimate partner violence, Egypt." Eastern Mediterranean Health Journal 27(3): 233-241. | R | Not Refugee Women |
| 74 | Idehen, E. E., T. Korhonen, A. Castaneda, T. Juntunen, M. Kangasniemi, A.-M. Pietilä and P. Koponen (2017). "Factors associated with cervical cancer screening participation among immigrants of Russian, Somali and Kurdish origin: a population-based study in Finland." BMC Women's Health 17: 1-10. | R | Not Refugee Women |
| 75 | Ikenberg, H., J. Obwegeser and V. Schneider (2006). "Who controls the controllers?...Obwegeser J, Schneider V. Thin-layer cervical cytology: a new meta-analysis. Lancet 2006; 367:88-89." Lancet 368(9535): 578-579. | R | Review Paper |
| 76 | Iqbal, J., O. Ginsburg, S. A. Narod, P. A. Rochon, H. D. Fischer, P. C. Austin and M. I. Creatore (2017). "A Population-Based Cross-Sectional Study Comparing Breast Cancer Stage at Diagnosis between Immigrant and Canadian-Born Women in Ontario." Breast Journal 23(5): 525-536. | R | Wrong Outcome |
| 77 | Irandoost, S. F., J. Yoosefi Lebni, H. Safari, F. Khorami, S. Ahmadi, G. Soofizad and F. Ebadi Fard Azar (2022). "Explaining the challenges and adaptation strategies of nurses in caring for patients with COVID-19: a qualitative study in Iran." BMC Nursing 21(1): 1-16. | R | Not Refugee Women |
| 78 | Jannati, A., S. H. Ebrahimi and M. Azizzadeh (2021). "Challenges and Obstacles to Clinical Guidelines from the Perspectives of Urology Residents: A Qualitative Study." Depiction of Health 11(4): 316-323. | R | Wrong Outcome |
| 79 | Jennings, B. M., M. Lem, S. Kilborn, B. Donnelly and A. Acker (2022). "Improving oral health care accessibility for homeless and vulnerably housed pet-owning populations." Canadian Journal of Dental Hygiene 56(2): 99-103. | R | Wrong Outcome |
| 80 | Jensen, J. D., M. Pokharel, A. J. King, K. K. John, Y. P. Wu and D. Grossman (2020). "Obstacles to skin self-examination: are frontier adults inclined abstainers?" Psychology, Health & Medicine 25(4): 470-479. | R | Wrong Outcome |
| 81 | Jia Lu Lilian, L. I. N., S. Quartaron, N. Aidaru, C. Y. Chan, J. Hubbert, J. Orkin, N. Fayed, N. Major, J. Soscia, A. Lim, S. D. French, M. E. Moretti and E. Cohen (2021). "Process Evaluation of a Hub-and-Spoke Model to Deliver Coordinated Care for Children with Medical Complexity across Ontario: Facilitators, Barriers and Lessons Learned." Healthcare Policy 17(1): 104-122. | R | Wrong Outcome |
| 82 | Jinshi, P. and E. S. Eunyoung (2022). "Predictive Factors of the Regular Mammography Screening among Korean Chinese Women in Korea." Asian Oncology Nursing 22(1): 56-64. | R | Not Refugee Women |
| 83 | Jusril, N. A., S. I. Abu Bakar, K. A. Khalil, W. M. Md Saad, N. K. Wen and M. I. Adenan (2022). "Development and Optimization of Nanoemulsion from Ethanolic Extract of Centella asiatica (NanoSECA) Using D-Optimal Mixture Design to Improve Blood-Brain Barrier Permeability." Evidence-based Complementary & Alternative Medicine (eCAM): 1-18. | R | Wrong Outcome |
| 84 | Kacperczyk-Bartnik, J., E. Bilir, O. Hoptyana, K. Zak, A. Shushkevich, A. Pletnev, Z. Razumova, A. Strojna, C. Theofanakis, N. Bizzarri and M. Bobinski (2023). "CERVICAL CANCER PREVENTION among UKRAINIAN REFUGEES during the FIRST SIX MONTHS after the BEGINNING of the WAR: The EUROPEAN NETWORK of YOUNG GYNAE ONCOLOGISTS (ENYGO) SURVEY RESULTS." International Journal of Gynecological Cancer 33(Supplement 3): A353. | R | Wrong Outcome |
| 85 | Kagawa-Singer, M., S. P. Tanjasiri, A. Valdez, H. Yu and M. A. Foo (2009). "Outcomes of a breast health project for Hmong women and men in California." American Journal of Public Health 99(S2): S467-473. | R | Wrong Outcome |
| 86 | Kamaraju, S., M. Denomie, A. Visotcky, A. Banerjee, K. Krause, E. Tavares, A. Rao, E. Drew, J. Neuner and M. Stolley (2018). "Increasing mammography uptake through academic-community partnerships targeting immigrant and refugee communities in Milwaukee." Wisconsin Medical Journal 117(2): 55-61. | R | Wrong Outcome |
| 87 | Kenny, D. X., K. Hsueh, R. W. Walters and J. J. Coté (2021). "Human Papillomavirus Vaccination and Pap Smear Rates Among Burmese Refugee Girls in a Healthcare System in Omaha, Nebraska." Journal of Community Health 46(6): 1170-1176. | R | Wrong Outcome |
| 88 | Kerans, M. (2004). "Breast cancer screening behavior among low-income and minority women." Clinical Excellence for Nurse Practitioners 8(1): 14-21. | R | Wrong Outcome |
| 89 | Khokhar, M. A., M. O. Niaz, A. Aslam, H. A. Khan, A. Loya, P. M. Speight and S. A. Khurram (2021). "Pakistan Oral Cancer Collaborative: analyzing barriers and obstacles to oral cancer diagnosis, treatment, and prevention in Pakistan." Oral Surgery, Oral Medicine, Oral Pathology & Oral Radiology 132(3): 312-319. | R | Not Refugee Women |
| 90 | Kim, K., S. Kim and Y. Chung (2017). "A qualitative study exploring factors associated with Pap test use among North Korean refugees." Health care for women international 38(10): 1115-1129. | A | - |
| 91 | Kim, K., S. Kim, J. J. Gallo, M. T. Nolan and H. R. Han (2017). "Decision making about Pap test use among Korean immigrant women: A qualitative study." Health Expectations 20(4): 685-695. | R | Wrong Outcome |
| 92 | King, E. M. and D. S. Busolo (2022). "The role of primary care Nurse Practitioners in reducing barriers to cervical cancer screening: A literature review." Canadian Oncology Nursing Journal 32(2): 233-257. | R | Review Paper |
| 93 | Kolar, M., I. Lukšič and B. Gabrovec (2021). "Public opinion on the eligibility of health care for migrants and refugees in Slovenia." Eastern Mediterranean Health Journal 27(12): 1182-1188. | R | Wrong Outcome |
| 94 | Kue, J., H. Hanegan and A. Tan (2017). "Perceptions of Cervical Cancer Screening, Screening Behavior, and Post-Migration Living Difficulties Among Bhutanese-Nepali Refugee Women in the United States." J Community Health 42(6): 1079-1089. | A | - |
| 95 | Kue, J., L. A. Szalacha, K. Rechenberg, T. S. Nolan and U. Menon (2021). "Communication Among Southeast Asian Mothers and Daughters About Cervical Cancer Prevention." Nursing research 70(5S Suppl 1): S73-S83. | R | Wrong Outcome |
| 96 | Kue, J., A. Zukoski, K. L. Keon and S. Thorburn (2014). "Breast and cervical cancer screening: exploring perceptions and barriers with Hmong women and men in Oregon." Ethnicity & Health 19(3): 311-327. | R | Not Refugee Women |
| 97 | Lai, D., J. Bodson, F. Davis, D. Lee, F. Tavake-Pasi, E. Napia, J. Villalta, V. Mukundente, R. Mooney, H. Coulter, L. Stark, A. Sanchez-Birkhead and D. Kepka (2017). "Diverse Families' Experiences with HPV Vaccine Information Sources: A Community-Based Participatory Approach." Journal of Community Health 42(2): 400-412. | R | Wrong Outcome |
| 98 | Lee, H. Y. and S. Vang (2010). "Barriers to cancer screening in Hmong Americans: the influence of health care accessibility, culture, and cancer literacy." Journal of Community Health 35(3): 302-314. | R | Not Refugee Women |
| 99 | Leinonen, M. K., S. Campbell, G. Ursin, A. Tropé and M. Nygård (2017). "Barriers to cervical cancer screening faced by immigrants: a registry-based study of 1.4 million women in Norway." European Journal of Public Health 27(5): 873-879. | R | Not Refugee Women |
| 100 | Lim, C., M. Hernandez, L. Gaona and C. Barrio (2021). "Recruitment of Asian Americans with Schizophrenia Spectrum Disorder for Research Participation: Barriers, Strategies, and Outcomes." Community Mental Health Journal 57(3): 490-501. | R | Wrong Outcome |
| 101 | Lofters, A. K., R. Moineddin, S. W. Hwang and R. H. Glazier (2011). "Predictors of low cervical cancer screening among immigrant women in Ontario, Canada." BMC Women's Health 11(1): 20-20. | R | Not Refugee Women |
| 102 | Lor, B., I. J. Ornelas, M. Magarati, H. H. Do, Y. Zhang, J. C. Jackson and V. M. Taylor (2018). "We Should Know Ourselves: Burmese and Bhutanese Refugee Women's Perspectives on Cervical Cancer Screening." Journal of health care for the poor and underserved 29(3): 881-897. | A | - |
| 103 | Luft, H., M. Perzan, R. Mitchell and A. Schmidt (2021). "An integrative literature review of barriers and facilitators to cervical cancer screening among refugee women in the United States." Health care for women international 42(7-9): 992-1012. | R | Review Paper |
| 104 | Lyons, G., S. Rengaswamy, A. B. Millar and S. Slama (2018). "Scaling up cancer care in the WHO Eastern Mediterranean Region." Eastern Mediterranean Health Journal 24(1): 104-110. | R | Wrong Outcome |
| 105 | Ma, G. X., M. Lee, L. Zhu, Y. Tan, P. Do, X. Ma, T. Tran and C. K. Johnson (2020). "Colorectal Cancer-Related Knowledge, Acculturation, and Healthy Lifestyle Behaviors Among Low-Income Vietnamese Americans in the Greater Philadelphia Metropolitan Area." Journal of Community Health 45(6): 1178-1186. | R | Wrong Outcome |
| 106 | Mahloch, J., J. C. Jackson, K. Chitnarong, R. Sam, L. S. Ngo and V. M. Taylor (1999). "Bridging cultures through the development of a cervical cancer screening video for Cambodian women in the United States." Journal of cancer education : the official journal of the American Association for Cancer Education 14(2): 109-114. | R | Wrong Outcome |
| 107 | Maiko, S., N. Hirokazu, B. Tomoya and I. Tomoyuki (2021). "Practical Challenges of Creating and Managing the "Barrier-Free Basic Plan" for Better Accessibility in Cities by Local Governments in Japan." Studies in Health Technology & Informatics(282): 333-347. | R | Not Refugee Women |
| 108 | Maleki, M., A. Mardani, M. Ghafourifard and M. Vaismoradi (2022). "Changes and challenges in sexual life experienced by the husbands of women with breast cancer: a qualitative study." BMC Women's Health 22(1): 1-11. | R | Wrong Outcome |
| 109 | Martinez, A. A., M. U. Malinverno, E. Manin, P. Petignat and J. Abdulcadir (2021). "A Cross-sectional Study on the Prevalence of Cervical Dysplasia among Women with Female Genital Mutilation/Cutting." Journal of Lower Genital Tract Disease 25(3): 210-215. | R | Wrong Outcome |
| 110 | McComb, E., V. Ramsden, O. Olatunbosun and H. Williams-Roberts (2018). "Knowledge, Attitudes and Barriers to Human Papillomavirus (HPV) Vaccine Uptake Among an Immigrant and Refugee Catch-Up Group in a Western Canadian Province." Journal of immigrant and minority health 20(6): 1424-1428. | R | Wrong Outcome |
| 111 | McLachlan, E., S. Anderson, D. Hawkes, M. Saville and K. Arabena (2018). "Ballarat and District Aboriginal Collective, Baarlinjan Medical Clinic, Ballarat, Victoria, Australia." Current Oncology 25(1): e17-e26. | R | Not Refugee Women |
| 112 | Meana, M., T. Bunston, U. George, L. Wells and W. Rosser (2001). "Influences on breast cancer screening behaviors in tamil immigrant women 50 years old and over." Ethnicity & Health 6(3/4): 179-188. | R | Wrong Outcome |
| 113 | Mekonnen, B. D., V. Vasilevski, A. G. Bali and L. Sweet (2024). "Effect of pregnancy intention on completion of maternity continuum of care in Sub-Saharan Africa: systematic review and meta-analysis." BMC Pregnancy and Childbirth 24(1): 802. | R | Wrong Outcome |
| 114 | Metusela, C., J. Ussher, J. Perz, A. Hawkey, M. Morrow, R. Narchal, J. Estoesta and M. Monteiro (2017). "'In My Culture, We Don't Know Anything About That': Sexual and Reproductive Health of Migrant and Refugee Women." International Journal of Behavioral Medicine 24(6): 836-845. | R | Wrong Outcome |
| 115 | Miesfeldt, S., C. Hayden, N. Apedoe, S. Jerome and A. Fletcher (2010). "Colorectal cancer screening pilot program for underserved women in Cumberland County, Maine." Journal of Community Health 35(2): 109-114. | R | Wrong Outcome |
| 116 | Miller, B. C., E. A. Sarma, Y. Sun, C. R. Messina and A. Moyer (2020). "Psychosocial predictors of mammography history among Chinese American women without a recent mammogram." Ethnicity & Health 25(6): 862-873. | R | Wrong Outcome |
| 117 | Missiakos, O., M. Brooks and A. Hawkey (2021). "A community-based health approach to increasing cervical screening among migrant and refugee women from an Arabic speaking background living in Western Sydney." Australian Journal of Primary Health 27(4): xxxiii. | R | Not Refugee Women |
| 118 | Mock, J., S. J. McPhee, T. Nguyen, C. Wong, H. Doan, K. Q. Lai, K. H. Nguyen, T. T. Nguyen and N. Bui-Tong (2007). "Effective lay health worker outreach and media-based education for promoting cervical cancer screening among Vietnamese American women." American Journal of Public Health 97(9): 1693-1700. | R | Wrong Outcome |
| 119 | Mofenson, L. M., M. T. Brady, S. P. Danner, K. L. Dominguez, R. Hazra, E. Handelsman, P. Havens, S. Nesheim, J. S. Read, L. Serchuck and R. Van Dyke (2009). "Guidelines for the prevention and treatment of opportunistic infections among HIV-exposed and HIV-infected children: recommendations from CDC, the National Institutes of Health, the HIV Medicine Association of the Infectious Diseases Society of America, the Pediatric Infectious Diseases Society, and the American Academy of Pediatrics." MMWR Recommendations & Reports 58(RR-11): 1-166. | R | Wrong Outcome |
| 120 | Murphy, J. E., C. Onwuzurike and A. Barden-Maja (2015). "A patient-centered appropach to addressing women's health in a refugee population." Journal of General Internal Medicine 30(SUPPL. 2): S520. | R | Wrong Outcome |
| 121 | Muz, G., G. E. Yüce, C. Yıldırım and M. Dağdelen (2021). "Obstacles and Related Factors Faced by Individuals with Type 2 Diabetes in Managing Diabetes." Journal of Education & Research in Nursing / Hemsirelikte Egitim ve Arastirma Dergisi 18(4): 389-395. | R | Wrong Outcome |
| 122 | Nagamatsu, Y., E. Barroga, Y. Sakyo, Y. Igarashi and Y. Hirano O (2020). "Risks and perception of non-communicable diseases and health promotion behavior of middle-aged female immigrants in Japan: a qualitative exploratory study." BMC Women's Health 20(1): 1-9. | R | Wrong Outcome |
| 123 | Navidian, A. and M. Sharmi (2017). "Evaluation of the Relationship between Post-traumatic Stress Disorder and Post-traumatic Growth in Women with Breast Cancer." Medical-Surgical Nursing Journal 6(2/3): 9-16. | R | Wrong Outcome |
| 124 | Nguyen, B. H., S. J. McPhee, S. L. Stewart and H. T. Doan (2010). "Effectiveness of a controlled trial to promote colorectal cancer screening in Vietnamese Americans." American Journal of Public Health 100(5): 870-876. | R | Wrong Outcome |
| 125 | Nguyen-Truong, C. K. Y., F. Lee-Lin, M. C. Leo, V. Gedaly-Duff, L. M. Nail, P.-r. Wang and T. Tran (2012). "A community-based participatory research approach to understanding Pap testing adherence among Vietnamese American immigrants." Journal of Obstetric, Gynecologic, & Neonatal Nursing: Clinical Scholarship for the Care of Women, Childbearing Families, & Newborns 41(6): E26-E40. | R | Wrong Outcome |
| 126 | Niyonsenga, G., D. Gishoma, R. Sego, M. G. Uwayezu, B. Nikuze, M. Fitch and P. C. Igiraneza (2021). "Knowledge, utilization and barriers of cervical cancer screening among women attending selected district hospitals in Kigali - Rwanda." Canadian Oncology Nursing Journal 31(3): 266-284. | R | Not Refugee Women |
| 127 | Norton, J. (2010). "Commissioning primary care services to improve sexual health." Practice Nurse 39(4): 36-42. | R | Wrong Outcome |
| 128 | Novotny, M., M. Kment and O. Viklicky (2021). "Antibody-Mediated Rejection of Renal Allografts: Diagnostic Pitfalls and Challenges." Physiological Research 70: S551-S565. | R | Wrong Outcome |
| 129 | Ogunsiji, O. O., C. Kwok, F. Lee Chun and L. C. Fan (2017). "Breast cancer screening practices of African migrant women in Australia: a descriptive cross-sectional study." BMC Women's Health 17: 1-10. | R | Wrong Outcome |
| 130 | Ornelas, I. J., K. Ho, J. C. Jackson, J. Moo-Young, A. Le, H. H. Do, B. Lor, M. Magarati, Y. Zhang and V. M. Taylor (2018). "Results From a Pilot Video Intervention to Increase Cervical Cancer Screening in Refugee Women." Health education & behavior : the official publication of the Society for Public Health Education 45(4): 559-568. | R | Wrong Outcome |
| 131 | Østergaard, L. S., M. Norredam, C. Mock-Munoz de Luna, M. Blair, S. Goldfeld and A. Hjern (2017). "Restricted health care entitlements for child migrants in Europe and Australia." European Journal of Public Health 27(5): 869-873. | R | Not Refugee Women |
| 132 | Ostrach, B. and M. Singer (2012). "At special risk: Biopolitical vulnerability and HIV/STI syndemics among women." Health Sociology Review 21(3): 258-271. | R | Wrong Outcome |
| 133 | Otoukesh, S., M. Mojtahedzadeh, R. A. Figlin, F. P. Rosenfelt, A. Behazin, D. Sherzai, C. J. Cooper and Z. A. Nahleh (2015). "Literature review and profile of cancer diseases among Afghan refugees in Iran: Referrals in six years of displacement." Medical Science Monitor 21: 3622-3628. | R | Review Paper |
| 134 | Ott, J. J., A. M. Paltiel and H. Becher (2009). "Noncommunicable disease mortality and life expectancy in immigrants to Israel from the former Soviet Union: country of origin compared with host country." Bulletin of the World Health Organization 87(1): 20-29. | R | Wrong Outcome |
| 135 | Özışık, P. A., K. M. Özdener, B. Ural, U. Er and A. Savaş (2022). "Clinical and ethical perspective of neurosurgical care in patients from beyond the southern border of Turkey: challenges of patients in war." Journal of Public Health (Germany) 30(6): 1431-1440. | R | Wrong Outcome |
| 136 | Pacik, D., D. W. Tolchin, R. Fortinsky and J. Robison (2022). "Moving Back Into the Community: Obstacles for People With an Acquired Brain Injury or Physical Disability." Journal of the American Medical Directors Association 23(8): 1396-1402. | R | Wrong Outcome |
| 137 | Paget, D. Z. (2014). "European Public Health News." European Journal of Public Health 24(1): 176-177. | R | Irrelevant |
| 138 | Pantha, S., M. J. Aguinaldo, S. M. Hasan-ul-Bari, S. Chowdhury, U. Dendup, R. D. Gupta, I. Sutradhar, R. Bari and M. Sarker (2022). "Facilitators and Barriers to Implementation of a Childhood Tuberculosis Control Program in Bangladesh: A Mixed-Methods Study from BRAC Urban DOTS Centres in Dhaka." Nursing Reports 12(2): 371-386. | R | Wrong Outcome |
| 139 | Parajuli, J., D. Horey and M.-I. Avgoulas (2020). "Perceived barriers to cervical cancer screening among refugee women after resettlement: A qualitative study." Contemporary nurse 56(4): 363-375. | A | - |
| 140 | Pentaris, P., D. Papadatou, A. Jones and G. M. Hosang (2018). "Palliative care professional’s perceptions of barriers and challenges to accessing children’s hospice and palliative care services in South East London: A preliminary study." Death Studies 42(10): 649-657. | R | Wrong Outcome |
| 141 | Patel, P., D. M. Muscat, L. Trevena, D. Zachariah, H. Nosir, N. Jesurasa, A. Hadi and S. Bernays (2022). "Exploring the expectations, experiences and tensions of refugee patients and general practitioners in the quality of care in general practice." Health Expectations 25(2): 639-647. | R | Wrong Outcome |
| 142 | Pentaris, P., D. Papadatou, A. Jones and G. M. Hosang (2018). "Palliative care professional’s perceptions of barriers and challenges to accessing children’s hospice and palliative care services in South East London: A preliminary study." Death Studies 42(10): 649-657. | R | Wrong Outcome |
| 143 | Percac-Lima, S., J. M. Ashburner, B. Bond, S. A. Oo and S. J. Atlas (2013). "Decreasing disparities in breast cancer screening in refugee women using culturally tailored patient navigation." Journal of General Internal Medicine 28(11): 1463-1468. | R | Wrong Outcome |
| 144 | Percac-Lima, S., B. Bond and A. Saadi (2013). "Bosnian, Iraqi, and somali refugee women speak: A comparative study of refugee health beliefs on preventive health and breast cancer screening." Journal of General Internal Medicine 28(SUPPL. 1): S35. | R | Wrong Outcome |
| 145 | Percac-Lima, S., B. Milosavljevic, S. A. Oo, D. Marable and B. Bond (2012). "Patient navigation to improve breast cancer screening in bosnian refugees and immigrants." Journal of Immigrant and Minority Health 14(4): 727-730. | R | Wrong Outcome |
| 146 | Persson, G., L. Barlow, A. Karlsson, M. Rosén, C. Stefansson, T. Theorell, P. Tüll and A. Aberg (2001). "Chapter 3. major health problems." Scandinavian Journal of Public Health 29(s58): 37-102. | R | Wrong Outcome |
| 147 | Phillips, S., S. Raskin, Y. Zhang and M. Pratt-Chapman (2020). "Perspectives from oncology patient navigation programs on information management practices and needs: a descriptive study." Supportive Care in Cancer 28(2): 515-524. | R | Wrong Outcome |
| 148 | Power, R., J. M. Ussher, A. Hawkey, O. Missiakos, J. Perz, O. Ogunsiji, N. Zonjic, C. Kwok, K. McBride and M. Monteiro (2022). "Co-designed, culturally tailored cervical screening education with migrant and refugee women in Australia: a feasibility study." BMC women's health 22(1): 353. | R | Wrong Outcome |
| 149 | Racine, L., I. Andsoy, S. Maposa, H. Vatanparast and S. Fowler-Kerry (2022). "Examination of Breast Cancer Screening Knowledge, Attitudes, and Beliefs among Syrian Refugee Women in a Western Canadian Province." The Canadian journal of nursing research = Revue canadienne de recherche en sciences infirmieres 54(2): 177-189. | R | Not Refugee Women |
| 150 | Racine, L. and I. Isik Andsoy (2022). "Barriers and Facilitators Influencing Arab Muslim Immigrant and Refugee Women’s Breast Cancer Screening: A Narrative Review." Journal of Transcultural Nursing 33(4): 542-549. | R | Review Paper |
| 151 | Ramsahoi, C. E., S. S. Sonny and J. M. Monk (2022). "Exploring Barriers to Food Security Among Immigrants: A Critical Role for Public Health Nutrition." Canadian Journal of Dietetic Practice & Research 83(2): 68-74. | R | Wrong Outcome |
| 152 | Ren, H., A. L. Wagner, J.-Y. Xie, K.-Y. Chen, Y.-H. Lu, X.-B. Zheng, T. Huang, M. L. Boulton and X.-X. Chen (2019). "How Do Experts and Nonexperts Want to Promote Vaccines? Hepatitis E Vaccine as Example." Health Services Insights 12: N.PAG-N.PAG. | R | Wrong Outcome |
| 153 | Resnicow, K., M. J. Stiffler and K. J. Ajrouch (2022). "Looking Back: The Contested Whiteness of Arab Identity." American Journal of Public Health 112(8): 1092-1096. | R | Irrelevant |
| 154 | Ricciardi, W., C. Signorelli and D. Z. Paget (2015). "8TH EUROPEAN PUBLIC HEALTH CONFERENCE." European Journal of Public Health 25(suppl_3): 2-476. | R | Irrelevant |
| 155 | Riley Risher, C., C. A. Hall, C. Skelly and B. Blair Brown (2021). "RN-BSN COMPLETION: BARRIERS AND CHALLENGES FACED BY AFRICAN AMERICAN NURSES." Journal of Cultural Diversity 28(3): 67-72. | R | Not Refugee Women |
| 156 | Rogan, J., M. Zielke, K. Drumright and L. M. Boehm (2020). "Institutional Challenges and Solutions to Evidence-Based, Patient-Centered Practice: Implementing ICU Diaries." Critical Care Nurse 40(5): 47-56. | R | Wrong Outcome |
| 157 | Rogers, C. R., O. J. Obidike, S. F. Wallington, M. Hussein, Z. A. Mahamed and J. Sampson (2021). "A qualitative study of barriers and enablers associated with colorectal cancer screening among Somali men in Minnesota." Ethnicity & Health 26(2): 168-185. | R | Wrong Outcome |
| 158 | Saad, F., M. Ayyash, M. Ayyash, N. Elhage, I. Ali, M. Makki, H. Hamade and R. A. Blackwood (2020). "Assessing Knowledge, Physician Interactions and Patient-Reported Barriers to Colorectal Cancer Screening Among Arab Americans in Dearborn, Michigan." Journal of Community Health 45(5): 900-909. | R | Wrong Outcome |
| 159 | Saadi, A., B. Bond and S. Percac-Lima (2012). "Perspectives on preventive health care and barriers to breast cancer screening among iraqi women refugees." Journal of Immigrant and Minority Health 14(4): 633-639. | R | Wrong Outcome |
| 160 | Saadi, A., B. E. Bond and S. Percac-Lima (2015). "Bosnian, Iraqi, and Somali Refugee Women Speak: A Comparative Qualitative Study of Refugee Health Beliefs on Preventive Health and Breast Cancer Screening." Women's Health Issues 25(5): 501-508. | R | Wrong Outcome |
| 161 | Sadeghi, N., S. A. Hossein Hesami, S. Sadeghi and M. Sadeghi (2021). "Barriers to Palliative Care in the Neonatal Intensive Care Unit from Nurses’ Perspective: A Qualitative Study." Medical-Surgical Nursing Journal 10(2): 1-8. | R | Wrong Outcome |
| 162 | Salman, K. F. (2012). "Health Beliefs and Practices Related to Cancer Screening Among Arab Muslim Women in an Urban Community." Health Care for Women International 33(1): 45-74. | R | Wrong Outcome |
| 163 | Sarikhani, Y., P. Bastani, M. Rafiee, Z. Kavosi and R. Ravangard (2021). "Key Barriers to the Provision and Utilization of Mental Health Services in Low-and Middle-Income Countries: A Scope Study." Community Mental Health Journal 57(5): 836-852. | R | Wrong Outcome |
| 164 | Sawyer, B., E. Hilliard, K. J. Hackney and S. Stastny (2022). "Barriers and Strategies for Type 1 Diabetes Management Among Emerging Adults: A Qualitative Study." Clinical Medicine Insights: Endocrinology & Diabetes: 1-10. | R | Wrong Outcome |
| 165 | Schrier, L., C. Wyder, S. del Torso, T. Stiris, U. von Both, J. Brandenberger and N. Ritz (2019). "Medical care for migrant children in Europe: a practical recommendation for first and follow-up appointments." European Journal of Pediatrics 178(9): 1449-1467. | R | Wrong Outcome |
| 166 | Schuster, R. C., E. M. Rodriguez, M. Blosser, A. Mongo, N. Delvecchio-Hitchcock, L. Kahn and L. Tumiel-Berhalter (2019). ""They were just waiting to die": Somali Bantu and Karen Experiences with Cancer Screening Pre- and Post-Resettlement in Buffalo, NY." Journal of the National Medical Association 111(3): 234-245. | R | Wrong Outcome |
| 167 | Shea, L., J. Pesa, G. Geonnotti, V. Powell, C. Kahn and W. Peters (2022). "Improving diversity in study participation: Patient perspectives on barriers, racial differences and the role of communities." Health Expectations 25(4): 1979-1987. | R | Wrong Outcome |
| 168 | Shirazi, M., A. Shirazi and J. Bloom (2015). "Developing a Culturally Competent Faith-Based Framework to Promote Breast Cancer Screening Among Afghan Immigrant Women." Journal of Religion and Health 54(1): 153-159. | R | Not Refugee Women |
| 169 | Shoemark, T. and P. Foran (2021). "CERNER LES OBSTACLES À LA DÉFENSE DES INTÉRÊTS DES PATIENTS DANS LA PROMOTION D'UNE CULTURE DE SÉCURITÉ : UNE REVUE INTÉGRATIVE." ORNAC Journal 39(4): 32-50. | R | Irrelevant |
| 170 | Spiegel, P. B., J. G. Cheaib, S. A. Aziz, O. Abrahim, M. Woodman, A. Khalifa, M. Jang and F. J. Mateen (2020). "Cancer in Syrian refugees in Jordan and Lebanon between 2015 and 2017." The Lancet Oncology 21(5): e280-e291. | R | Wrong Outcome |
| 171 | Stirling-Cameron, E., S. Almukhaini, J. Dol, B. J. DuPlessis, K. Stone, M. Aston and S. M. Goldenberg (2024). "Access and use of sexual and reproductive health services among asylum-seeking and refugee women in high-income countries: A scoping review." PLoS ONE 19(11 November): e0312746. | R | Wrong Outcome |
| 172 | Stodart, K. (2018). "Working for women's health." Kai Tiaki Nursing New Zealand 24(2): 18-19. | R | Irrelevant |
| 173 | Sweity, E. M., A. M. Salahat, A. a. Sada, A. Aswad, L. M. Zabin and S. e. H. Zyoud (2022). "Knowledge, attitude, practice and perceived barriers of nurses working in intensive care unit on pain management of critically ill patients: a cross-sectional study." BMC Nursing 21(1): 1-10. | R | Wrong Outcome |
| 174 | Tatari, C. R., B. Andersen, T. Brogaard, S. Badre‐Esfahani, N. Jaafar and P. Kirkegaard (2021). "The SWIM study: Ethnic minority women's ideas and preferences for a tailored intervention to promote national cancer screening programmes—A qualitative interview study." Health Expectations 24(5): 1692-1700. | R | Wrong Outcome |
| 175 | Tawiah, A. K., A. Borthwick and L. Woodhouse (2020). Advanced Physiotherapy Practice: A qualitative study on the potential challenges and barriers to implementation in Ghana. Philadelphia, Pennsylvania, Taylor & Francis Ltd. 36: 307-315. | R | Wrong Outcome |
| 176 | Taylor, V. M., J. C. Jackson, Y. Yasui, T. T. Nguyen, E. Woodall, E. Acorda, L. Li and S. Ramsey (2010). "Evaluation of a cervical cancer control intervention using lay health workers for Vietnamese American women." American Journal of Public Health 100(10): 1924-1929. | R | Wrong Outcome |
| 177 | Thiel de Bocanegra, H., Z. Goliaei, N. Khan, S. Banna, R. Behnam and S. K. Mody (2022). "Refugee Women's Receptiveness for Virtual Engagement on Reproductive Health During the COVID-19 Pandemic." International journal of behavioral medicine. | R | Wrong Outcome |
| 178 | Thiel de Bocanegra, H., Z. Goliaei, N. Khan, S. Banna, R. Behnam and S. K. Mody (2023). "Refugee Women's Receptiveness for Virtual Engagement on Reproductive Health During the COVID-19 Pandemic." International journal of behavioral medicine 30(3): 366-375. | R | Wrong Outcome |
| 179 | Thorburn, S., J. Kue, K. Keon and P. Lo (2012). "Medical Mistrust and Discrimination in Health Care: A Qualitative Study of Hmong Women and Men." Journal of Community Health 37(4): 822-829. | R | Wrong Outcome |
| 180 | Tindall, B. and D. Miller (1992). "Amsterdam conference review." AIDS Care 4(4): 425-425. | R | Irrelevant |
| 181 | Torres, S., R. LabontÉ, D. L. Spitzer, C. Andrew and C. Amaratunga (2014). "Improving Health Equity: The Promising Role of Community Health Workers in Canada." Healthcare Policy 10(1): 73-85. | R | Irrelevant |
| 182 | Vahabi, M., A. K. Lofters, A. Kopp and R. H. Glazier (2021). "Correlates of non-adherence to breast, cervical, and colorectal cancer screening among screen-eligible women: a population-based cohort study in Ontario, Canada." Cancer Causes & Control 32(2): 147-155. | R | Wrong Outcome |
| 183 | Valizadeh, L., V. Zamanzadeh, S. Saber and T. Kianian (2018). "Challenges and Barriers Faced by Home Care Centers: An Integrative Review." Medical-Surgical Nursing Journal 7(3): 1-9. | R | Review Paper |
| 184 | Wang, A. M. Q., E. M. Yung, N. Nitti, Y. Shakya, A. K. M. Alamgir and A. K. Lofters (2019). "Breast and colorectal cancer screening barriers among immigrants and refugees: A mixed-methods study at three community health centres in Toronto, Canada." Journal of Immigrant and Minority Health 21(3): 473-482. | R | Wrong Outcome |
| 185 | Watanabe-Galloway, S., N. Alnaji, B. Grimm and M. Leypoldt (2018). "Cancer Community Education in Somali Refugees in Nebraska." Journal of Community Health 43(5): 929-936. | R | Wrong Outcome |
| 186 | Whalen-Browne, M., R. Talavlikar, G. Brown, K. McBrien, M. L. Wiedmeyer, E. Norrie and G. Fabreau (2022). "Cervical Cancer Screening by Refugee Category in a Refugee Health Primary Care Clinic in Calgary, Canada, 2011-2016." Journal of immigrant and minority health. | R | Wrong Outcome |
| 187 | Wilson, L., T. Rubens-Augustson, M. Murphy, C. Jardine, N. Crowcroft, C. Hui and K. Wilson (2018). "Barriers to immunization among newcomers: A systematic review." Vaccine 36(8): 1055-1062. | R | Review Paper |
| 188 | Wilson, L. A., A. M. L. Quan, A. B. Bota, S. S. Mithani, M. Paradis, C. Jardine, C. Hui, K. Pottie, N. Crowcroft and K. Wilson (2021). "Newcomer knowledge, attitudes, and beliefs about human papillomavirus (HPV) vaccination." BMC family practice 22(1): 17. | R | Wrong Outcome |
| 189 | Woldetsadik, A. B., A. F. Amhare, S. T. Bitew, L. Pei, J. Lei and J. Han (2020). "Socio-demographic characteristics and associated factors influencing cervical cancer screening among women attending in St. Paul's Teaching and Referral Hospital, Ethiopia." BMC Women's Health 20(1): 1-9. | R | Not Refugee Women |
| 190 | Wu, T.-Y. and V. Raghunathan (2020). "Predictors of Preventive Health Practices, Chronic Disease Burden and Health Status Among Underserved Bangladeshi Americans in Michigan." Journal of Community Health 45(2): 310-318. | R | Not Refugee Women |
| 191 | Yarbro, C. H. (2003). "International nursing and breast cancer." Breast Journal 9: S98-100. | R | Irrelevant |
| 192 | Yelverton, V., S. Qiao, S. Weissman, B. Olatosi and X. Li (2021). "Telehealth for HIV Care Services in South Carolina: Utilization, Barriers, and Promotion Strategies During the COVID-19 Pandemic." AIDS & Behavior 25(12): 3909-3921. | R | Wrong Outcome |
| 193 | Yi, J., K. Anderson, Y.-C. Le, S. Escobar-Chaves and C. Reyes-Gibby (2013). "English Proficiency, Knowledge, and Receipt of HPV Vaccine in Vietnamese-American Women." Journal of Community Health 38(5): 805-811. | R | Not Refugee Women |
| 194 | Yixi, L. and L. Racine (2015). "Reviewing Chinese immigrant women's health experiences in English-speaking Western Countries: a postcolonial feminist analysis." Health Sociology Review 24(1): 15-28. | R | Not Refugee Women |
| 195 | Youngson, N., M. Saxton, P. G. Jaffe, D. Chiodo, M. Dawson and A.-L. Straatman (2021). "Challenges in Risk Assessment with Rural Domestic Violence Victims: Implications for Practice." Journal of Family Violence 36(5): 537-550. | R | Irrelevant |
| 196 | Zha, N., M. Alabousi, B. K. Patel and M. N. Patlas (2019). "Beyond Universal Health Care: Barriers to Breast Cancer Screening Participation in Canada." Journal of the American College of Radiology 16(4): 570-579. | R | Not Refugee Women |
| 197 | Zhang, Y., I. Ornelas, H. Do, M. Magarati, J. Jackson and V. Taylor (2017). "Provider Perspectives on Promoting Cervical Cancer Screening Among Refugee Women." Journal of Community Health 42(3): 583-590. | R | Not Refugee Women |
| 198 | Zhang, Y., I. J. Ornelas, H. H. Do, M. Magarati, J. C. Jackson and V. M. Taylor (2018). "Provider Perspectives on Promoting Cervical Cancer Screening Among Refugee Women." Journal of community health 42(3): 583-590. | R | Not Refugee Women |
| 199 | Adopting self-care interventions for sexual and reproductive health in the Eastern Mediterranean Region, World Health Organization. | R | Irrelevant |
| 200 | Meeting of the Strategic Advisory Group of Experts on Immunization, April 2022: conclusions and recommendations, World Health Organization. | R | Irrelevant |
| 201 | (1998). "Cultural and ethnic diversity." Health & Social Work 23(2): 83-158. | R | Irrelevant |
| 202 | (1998). "Imported Dracunculiasis -- United States, 1995 and 1997." MMWR: Morbidity & Mortality Weekly Report 47(11): 209-211. | R | Irrelevant |
| 203 | (1998). "Preventing emerging infectious diseases: a strategy for the 21st century. Overview of the updated CDC plan." MMWR: Morbidity & Mortality Weekly Report 47(35): 1-14. | R | Irrelevant |
| 204 | (1998). "Strategies for providing follow-up and treatment services in the National Breast and Cervical Cancer Early Detection Program -- United States, 1997." MMWR: Morbidity & Mortality Weekly Report 47(11): 215-218. | R | Irrelevant |
| 205 | (1998). "Update: HIV counseling and testing using rapid tests -- United States, 1995." MMWR: Morbidity & Mortality Weekly Report 47(11): 211-215. | R | Irrelevant |
| 206 | (1999). "Preventing emerging infectious diseases: a strategy for the 21st century." Journal of Environmental Health 61(6): 37-37. | R | Irrelevant |
| 207 | (2000). "Escherichia coli O111:HB outbreak among teenage campers -- Texas, 1999." MMWR: Morbidity & Mortality Weekly Report 49(15): 321-324. | R | Irrelevant |
| 208 | (2000). "HIV-related tuberculosis in a transgender network -- Baltimore, Maryland, and New York City area, 1998-2000." MMWR: Morbidity & Mortality Weekly Report 49(15): 317-320. | R | Irrelevant |
| 209 | (2000). "Prevalence of leisure-time physical activity among overweight adults -- United States, 1998." MMWR: Morbidity & Mortality Weekly Report 49(15): 326-330. | R | Irrelevant |
| 210 | (2000). "Public health aspects of the Rainbow Family of Living Light annual gathering -- Allegheny National Forest, Pennsylvania, 1999." MMWR: Morbidity & Mortality Weekly Report 49(15): 324-326. | R | Irrelevant |
| 211 | (2001). "Health on equal terms -- national goals for public health: final report by the Swedish National Committee for Public Health." Scandinavian Journal of Public Health 29(s57): 5-68. | R | Irrelevant |
| 212 | (2001). "National goals for public health." Scandinavian Journal of Public Health 29(s57): 20-66. | R | Irrelevant |
| 213 | (2006). "Conclusions and recommendations from the Immunization Strategic Advisory Group." Weekly Epidemiological Record 81(1): 2-11. | R | Irrelevant |
| 214 | (2007). "15th EUPHA conference. The future of public health in the Unified Europe: Helsinki, 11-13 October 2007." European Journal of Public Health 17: 1-240. | R | Conference Paper |
| 215 | (2011). "B. Interactive poster presentations." Psychology & Health 26: 73-253. | R | Conference Paper |
| 216 | (2012). "Oncology Nursing Society 37th Annual Congress Podium and Poster Abstracts." Oncology Nursing Forum 39(3): E157-225. | R | Conference Paper |
| 217 | (2013). "Abstracts for the International Symposium on Dental Hygiene, Cape Town, South Africa, August 14-17, 2013." International Journal of Dental Hygiene 11(3): 156-173. | R | Conference Paper |
| 218 | (2013). "EHPS 2013 Abstracts." Psychology & Health 28: 1-344. | R | Conference Paper |
| 219 | (2013). "Index to Volume 137 January through December 2013." Archives of Pathology & Laboratory Medicine 137(12): 1845-1881. | R | Irrelevant |
| 220 | (2013). "SUBJECT INDEX TO VOLUME 137." Archives of Pathology & Laboratory Medicine 137(12): 1852-1881. | R | Irrelevant |
| 221 | (2014). "115th Annual Meeting of the American Associaton of Colleges of Pharmacy, Grapevine, TX, July 26-30, 2014." American Journal of Pharmaceutical Education 78(5): 1-140. | R | Irrelevant |
| 222 | (2014). "News." Journal of Global Health 4(1): 010201-010201. | R | Irrelevant |
| 223 | (2014). "PARALLEL SESSION 2...7th European Public Health Conference, “Mind the Gap: Reducing inequalities in health and health care” November 2014, Glasgow." European Journal of Public Health 24(suppl_2): 52-110. | R | Conference Paper |
| 224 | (2015). Bibliography. Philadelphia, Pennsylvania, Taylor & Francis Ltd. 23: 169-197. | R | Irrelevant |
| 225 | (2016). "9th European Public Health Conference: Parallel Sessions." European Journal of Public Health 26: 4-495. | R | Conference Paper |
| 226 | (2017). "10TH EUROPEAN PUBLIC HEALTH CONFERENCE...10th European Public Health (EPH) Conference, 1 - 4 November 2017 in Stockholm, Sweden." European Journal of Public Health 27: 1-529. | R | Conference Paper |
| 227 | (2017). "2017 Oncology Nursing Society Annual Congress Podium and Poster Abstracts." Oncology Nursing Forum 44(2): 1-215. | R | Conference Paper |
| 228 | (2017). "Abstracts : 29th European Congress of Pathology." Virchows Archiv 471: 1-352. | R | Conference Paper |
| 229 | (2017). "Vocabulary." Taber's Cyclopedic Medical Dictionary (2017) 23rd Edition: 1-2555. | R | Irrelevant |
| 230 | (2018). "Abstract 2018...15th International Congress of Behavioral Medicine will take place in Santiago, Chile from November 14th to November 17th, 2018." International Journal of Behavioral Medicine 25(1): 1-219. | R | Conference Paper |
| 231 | (2018). "PARALLEL PROGRAMME...11th European Public Health Conference, Cankarjev Dom, Ljubljana, Slovenia." European Journal of Public Health 28: 5-308. | R | Irrelevant |
| 232 | (2018). "POSTER DISPLAYS...11th European Public Health Conference, November 28-December 1, 2018, Ljubljana, Slovenia." European Journal of Public Health 28: 409-512. | R | Irrelevant |
| 233 | (2018). "Poster Presentation Abstracts...25th Silver Jubilee International Conference of Indian Association of Palliative Care (IAPCON 2018), AIIMS, New Delhi, February 23 to 25, 2018." Indian Journal of Palliative Care 24(2): 228-282. | R | Irrelevant |
| 234 | (2018). "POSTER WALKS...11th European Public Health Conference, 28 November – 1 December 2018, Ljubljana, Slovenia." European Journal of Public Health 28: 309-408. | R | Irrelevant |
| 235 | (2019). "12th European Public Health Conference Building bridges for solidarity and public health Marseille, France 20th–23rd November 2019." European Journal of Public Health 29: 1-2305. | R | Conference Paper |
| 236 | (2019). "12 Interactive poster presentations." Psychology & Health 26: 73-253. | R | Conference Paper |
| 237 | (2019). "Abstracts Presented at the 120th Annual Meeting of the American Association of Colleges of Pharmacy, Chicago, Illinois, July 13-17, 2019." American Journal of Pharmaceutical Education 83(5): 908-1117. | R | Conference Paper |
| 238 | (2019). "CDHA 2019 NATIONAL CONFERENCE: PEER-REVIEWED PRESENTATIONS...October 3-5, St. John’s, Newfoundland & Labrador." Canadian Journal of Dental Hygiene 53(3): 183-188. | R | Conference Paper |
| 239 | (2019). "EAP 2019 Congress and Master Course." European Journal of Pediatrics 178(11): 1613-1800. | R | Irrelevant |
| 240 | (2020). "DS NON-COMMUNICABLE DISEASES (INCLUDING CANCER, CVD, DIABETES, ORAL HEALTH) .16th World Congress on Public Health (WCPH2020), (virtual congress), 12–16 October, 2020." European Journal of Public Health 30: v932-v964. | R | Irrelevant |
| 241 | (2021). "14th European Public Health Conference 2021...14th European Public Health Conference (Virtual), Public health futures in a changing world, November 10-12, 2021." European Journal of Public Health 31: iii1-iii3. | R | Irrelevant |
| 242 | (2022). "Public health round-up." Bulletin of the World Health Organization 100(5): 296-297. | R | Irrelevant |

*Note: A – Accepted; R – Rejected

**Table C: Reasons for exclusion of studies after full text review**

| **Author, Year and Country** | **Title of the Article** | **Reasons for Exclusion** |
| --- | --- | --- |
| Abboud et al; 2017 | Cervical Cancer Screening Among Arab Women in the United States: An Integrative Review. | Not on refugee women |
| Abdi et. al., (2020) | Cervical Cancer Screening Among Immigrant and Refugee Women: Scoping-Review and Directions for Future Research | Review Paper |
| Al Qadire et al,2020 | Syrian Refugees in Jordan: Barriers to receiving optimal cancer care. | Wrong outcomes (Cancer care and treatment and not specific to cervical cancer) |
| Alam Z et al., (2023) | Interventions to increase cervical screening uptake among immigrant women: A systematic review and meta-analysis. | Review Paper |
| Appel, H.; Cook, K. et al, 2017 | Cancer screening for immigrants and refugees- a cultural and linguistic approach. | Not on refugee women |
| Chongsuwat et al, (2024). | Development of Cervical Cancer Prevention Workshops for Hmong and Karenni Women Through a Community-Academic Partnership. | Wrong Outcomes (No barriers identified) |
| Church et al, (2023). | Student midwives’ perspectives of women’s sexual and reproductive health literacy in Turkey. | Not on refugee women |
| Fang & Baker (2013) | Barriers and facilitators of cervical cancer screening among women of Hmong origin | Not on refugee women (among women of Hmong origin) |
| Galanis, Petros et al,2022 | Healthcare Services Access, Use and Barriers among Migrants in Europe: A Systematic Review. | Not on refugee women |
| Kenny et al., (2021) | Human Papillomavirus Vaccination and Pap Smear Rates Among Burmese Refugee Girls in a Healthcare System in Omaha, Nebraska | Wrong Outcomes (No barriers identified) |
| Luft et al., (2021) | An integrative literature review of barriers and facilitators to cervical cancer screening among refugee women in the United States | Review Paper |
| McComb, Erin et al; 2018 | Knowledge, Attitudes and Barriers to Human Papillomavirus (HPV) Vaccine Uptake Among an Immigrant and Refugee Catch-Up Group in a Western Canadian Province. | Wrong outcomes (Knowledge, attitudes, and barriers to HPV) |
| Missiakos, O. et al; 2021 | A community-based health approach to increasing cervical screening among migrant and refugee women from an Arabic speaking background living in Western Sydney. | Not on refugee women |
| Schuster, R. C. et al,2019 | “They were just waiting to die”: Somali Bantu and Karen Experiences with Cancer Screening Pre- and Post-Resettlement in Buffalo, NY. | Not specific to cervical cancer |
| Stirling-Cameron et al, (2024). | Access and use of sexual and reproductive health services among asylum-seeking and refugee women in high-income countries: A scoping review | Review Paper |
| Thiel de Bocanegra et al, (2023) | Refugee Women’s Receptiveness for Virtual Engagement on Reproductive Health During the COVID‑19 Pandemic. | Wrong Outcome (Not on cervical cancer) |
| Whalen-Browne et al. (2022) | Cervical Cancer Screening by Refugee Category in a Refugee Health Primary Care Clinic in Calgary, Canada, 2011-2016 | Wrong Outcomes (No barriers identified) |
| Wilson, L. A. et al; 2021 | Newcomer knowledge, attitudes, and beliefs about human papillomavirus (HPV) vaccination. | Wrong outcomes (Newcomers’ Knowledge, attitudes, and beliefs on HPV) |

**Table D: Risk-of-bias of included qualitative studies**

| **Author, Year & Country** | **Clear statement of aims** | **Are qualitative methods appropriate** | **Research design appropriate to answer questions** | **Recruitment strategy appropriate** | **Data collection addresses the research issue** | **Relationship between researcher and participants** | **Ethical issues considered** | **Data analysis rigorous** | **Clear statement of findings** | **Value of the research** | **SUM of Scores** | **Quality score** |
| --- | --- | --- | --- | --- | --- | --- | --- | --- | --- | --- | --- | --- |
|  | *(Importance and relevance)* | *(Seeks to illuminate subjective experiences)* | *(Justify research design)* | *(Justify why the participants selected are suitable)* | *(Data collection methods explicit and justified)* | *(Critical examination of how researcher may bias results)* | *(Was there adequate consenting, confidentiality, and ethical review)* | *(How are presented data selected from the original transcripts)* | *(Is there discussion of data validity)* | *(Contribution the study makes to existing knowledge)* |  | 0-20 (0-10 low; 11-15 medium; 16-20 High) |
| Abdullahi et al. 2009 UK | 1 | 2 | 1 | 2 | 1 | 1 | 2 | 2 | 1 | 1 | 14 | Medium 14/20 |
| Haworth et al. 2014 USA | 2 | 2 | 2 | 2 | 1 | 2 | 1 | 1 | 1 | 1 | 15 | Medium 15/20 |
| Kim et al. 2017 South Korea | 2 | 2 | 1 | 1 | 1 | 1 | 2 | 2 | 2 | 1 | 15 | Medium 15/20 |
| Lor et al. 2018 USA | 2 | 2 | 2 | 1 | 2 | 1 | 2 | 1 | 1 | 1 | 15 | Medium 15/20 |
| Allen et al. 2019 USA | 1 | 1 | 1 | 1 | 1 | 1 | 1 | 1 | 1 | 1 | 10 | Poor 10/20 |
| Parajuli et al. 2020 Australia | 2 | 2 | 2 | 2 | 2 | 2 | 2 | 2 | 2 | 1 | 19 | High 19/20 |
| Ghebrendrias et al. 2021 USA | 2 | 2 | 2 | 1 | 1 | 1 | 1 | 1 | 1 | 1 | 13 | Medium 13/20 |

**Table E: Risk-of-bias of included quantitative studies**

| **Author, Year & Country** | **Clear statement of aims** | **Methodology** | **Study population** | **Confounding and biases** | **Reliability of the results** | **Tables and graphs** | **Statistical methods** | **Use of appropriate variables** | **Relevance of the study** | **SUM of Scores** | **Quality score** |
| --- | --- | --- | --- | --- | --- | --- | --- | --- | --- | --- | --- |
|  | *(Importance & relevance)* | *(An appropriate method to the study questions)* | *(An appropriate study design, sample size & power calculation)* | *(Including response rate & questionnaire validation)* | *(Has the study design flawed the results?)* | *(Are tables & graphs adequately labeled?)* | *(Are appropriate statistical methods employed?)* | *(Are appropriate statistical methods employed?)* | *(Can the results be applied to the local situation?)* |  | 0-18 (0-9 low; 10-14 medium; 15-18 High) |
| Kue et al. 2017 USA | 2 | 1 | 1 | 1 | 2 | 2 | 2 | 2 | 0 | 13 | Medium 13/18 |
| Muhaidat et al., 2022, Jordan | 2 | 2 | 2 | 0 | 1 | 1 | 1 | 1 | 2 | 12 | Medium 12/18 |
| Elmore et al., 2022, United States | 2 | 2 | 2 | 1 | 2 | 2 | 2 | 1 | 2 | 16 | High 18/18 |
| Al Abdul Kader et al., 2023 USA | 2 | 2 | 2 | 0 | 1 | 1 | 1 | 2 | 2 | 13 | Medium 13/18 |
